# Using Online Media to Increase the Awareness and Uptake of Preexposure Prophylaxis for HIV Among Asian-Born Men Who Have Sex With Men Living in Australia: An Open-Label Randomized Controlled Trial

**DOI:** 10.1093/ofid/ofaf321

**Published:** 2025-07-23

**Authors:** Warittha Tieosapjaroen, Tiffany R Phillips, Eric P F Chow, Christopher K Fairley, Satrio Nindyo Istiko, Jason Wu, James Tapa, Limin Mao, Lei Zhang, David Wang, Budiadi Sudarto, Jason J Ong

**Affiliations:** School of Translational Medicine, Faculty of Medicine, Nursing and Health Sciences, Monash University, Melbourne, Australia; Melbourne Sexual Health Centre, Alfred Health, Melbourne, Australia; School of Translational Medicine, Faculty of Medicine, Nursing and Health Sciences, Monash University, Melbourne, Australia; Melbourne Sexual Health Centre, Alfred Health, Melbourne, Australia; School of Translational Medicine, Faculty of Medicine, Nursing and Health Sciences, Monash University, Melbourne, Australia; Melbourne Sexual Health Centre, Alfred Health, Melbourne, Australia; Centre for Epidemiology and Biostatistics, Melbourne School of Population and Global Health, The University of Melbourne, Melbourne, Australia; School of Translational Medicine, Faculty of Medicine, Nursing and Health Sciences, Monash University, Melbourne, Australia; Melbourne Sexual Health Centre, Alfred Health, Melbourne, Australia; Bradford Institute for Health Research, Bradford Teaching Hospitals NHS Foundation Trust, Bradford, UK; Bristol Medical School, University of Bristol, Bristol, UK; School of Public Health, Faculty of Medicine, The University of Queensland, Brisbane, Australia; Melbourne Sexual Health Centre, Alfred Health, Melbourne, Australia; Kings Park Medical Centre–Hillside, General Practice, Melbourne, Australia; School of Translational Medicine, Faculty of Medicine, Nursing and Health Sciences, Monash University, Melbourne, Australia; Centre for Social Research in Health, University of New South Wales, Sydney, Australia; School of Translational Medicine, Faculty of Medicine, Nursing and Health Sciences, Monash University, Melbourne, Australia; Melbourne Sexual Health Centre, Alfred Health, Melbourne, Australia; Disease Elimination Program, Public Health Discipline, Burnet Institute, Melbourne, Australia; School of Translational Medicine, Faculty of Medicine, Nursing and Health Sciences, Monash University, Melbourne, Australia; School of Translational Medicine, Faculty of Medicine, Nursing and Health Sciences, Monash University, Melbourne, Australia; Melbourne Sexual Health Centre, Alfred Health, Melbourne, Australia; Faculty of Infectious and Tropical Diseases, London School of Hygiene and Tropical Medicine, London, UK

**Keywords:** crowdsourcing, men who have sex with men, migrant, preexposure prophylaxis for HIV, randomized controlled trial

## Abstract

**Background:**

HIV notifications have increased among Asian-born men who have sex with men (MSM) in Australia. However, most existing campaigns were not designed for this population. This study assessed the acceptability of using a community-based audio drama from our previous “designathon” to increase the awareness and uptake of preexposure prophylaxis (PrEP), as compared with written information on the PAN website (PrEP Access Now; PAN.org.au), among Asian-born MSM in Australia.

**Methods:**

This open-label, 2-arm pilot trial based on a stratified randomized controlled design was conducted between April and July 2024. A total of 200 Asian-born MSM were randomized 1:1 to receive either the *Hot Peach Tea* audio drama (intervention)—which featured a PrEP journey of an Asian-born man in the MSM community who had newly arrived in Australia—or the PrEP Access Now website (control), which is often suggested by clinicians in Victoria to gain PrEP-related information. An intention-to-treat analysis was used to evaluate the data. The primary outcomes included acceptability, attitudes toward PrEP, and intention to use PrEP. The secondary outcomes included PrEP initiation and adherence to PrEP and PrEP-related knowledge.

**Results:**

Among the 200 participants, 96 formed the control arm, and 104 formed the intervention arm. Extreme satisfaction was expressed by 74% (71/96) and 77% (80/104) of the control and intervention arms, respectively. Full engagement (ie, completed the online media) occurred in 22% (21/96) and 85% (88/104) of the control and intervention arms (*P* < .001). The median score increases in having a positive attitude toward PrEP were 4 (IQR, 0–7) and 7 (IQR, 3–12.5) in the control and intervention arms (*P* = .023). Both online media increased the uptake of PrEP, with no significant differences observed between the arms. At the 2-month follow-up, 18% (17/96) and 22% (23/104) initiated PrEP in the control and intervention arms. The most reported reasons for not initiating PrEP were concern about side effects (20%, 19/96) in the control arm and perceived inconvenience of PrEP use (16%, 17/104) in the intervention arm. Six participants in the control arm and 4 in the intervention arm discontinued PrEP.

**Conclusions:**

The *Hot Peach Tea* audio drama effectively engaged Asian-born MSM and positively influenced their attitudes toward PrEP use. Strategies to link individuals to care after the audio drama are needed to increase the uptake of PrEP. Future trials should consider incorporating further support in PrEP initiation for this population.

**Clinical Trials Registration:**

ACTRN12623001361695 (Australian New Zealand Clinical Trials Registry https://www.anzctr.org.au/Trial/Registration/TrialReview.aspx?id = 387003).

Preexposure prophylaxis (PrEP) is a safe and highly effective HIV biomedical prevention method [1]. Since the introduction of PrEP in 2016 [2, 3], HIV notifications among Australian-born MSM have declined significantly. However, in 2023, the number of new HIV cases among overseas-born individuals surpassed those of Australian-born individuals [4]. Specifically, annual HIV notifications increased from 170 in 2014 to 227 in 2023 among Asian-born men who have sex with men (MSM) [5]. In a subanalysis of a community-based cross-sectional survey for Asian MSM in Sydney and Melbourne, despite an increase in PrEP uptake among Asian MSM [6], a small group of Asian MSM who were born in Asia and were at higher risk of HIV infection were less likely to use PrEP. This group included those who had newly arrived, had limited English, and had condomless anal sex with a casual sex partner in the last 6 months [7]. In a 2021 cross-sectional survey of migrants living in Queensland, Australia, only 13% (27/208) of migrants from Southeast Asia and East Asia were aware of PrEP [8].

Despite a demand for PrEP among Asian-born MSM, additional barriers to PrEP access have been reported [9, 10]. Two qualitative studies on PrEP accessibility demonstrated that Asian-born MSM did not use PrEP due to following: insufficient knowledge of sexually transmitted infections (STIs), HIV, and PrEP in their countries of origin; the unaffordability of PrEP without government subsidies from the host country; challenges in navigating the health care system, Medicare, and the private health insurance system in Australia; and self-perceptions within their communities (ie, the belief that PrEP is for a high sexually active person, a stereotype that may not align with Asian cultural norm) [11, 12]. Additionally, most existing educational programs about PrEP do not target Asian-born MSM. In Victoria, clinicians usually advise Medicare-ineligible individuals who are interested in using PrEP to purchase PrEP online and visit the PAN website (PrEP Access Now; PAN.org.au), which provides PrEP-related information, such as how to initiate and purchase PrEP with or without Medicare.

To address these barriers, we conducted a “designathon” in April 2023, bringing together the community to codevelop solutions aimed at increasing the awareness and uptake of PrEP among Asian-born MSM. A designathon is a participatory approach where interdisciplinary participants, including end users, collaborate over a short period to develop ideas or prototypes to solve specific problems. These solutions are subsequently evaluated by topic experts or community partners before being implemented in a trial or real-world settings [13]. Designathons enhance a sense of community empowerment and ownership of the solution, leading to sustainable and impactful changes that address community needs [14]. In our designathon, 8 ideas were developed on the basis of ideas from our crowdsourcing open call [15] and presented to a judging panel, and the winning idea was an audio drama titled *Hot Peach Tea*. This audio drama features 3 queer characters representing Asian-born MSM at different life stages and PrEP experiences.

Digital storytelling interventions have been shown to effectively improve HIV-related knowledge and awareness in randomized controlled trials (RCTs) [16–18] and among migrants [19]. In 2 RCTs in Nigeria, secondary school students and adolescents who received an 8-week, twice-per-week digital storytelling intervention demonstrated significant improvements in HIV knowledge and perceived risk of HIV/AIDS as measured by the HIV Knowledge Questionnaire–18 and the Perceived Risk of HIV Scale [17, 18]. Similarly, in an RCT of gay, bisexual, and other MSM in Singapore, a web drama video series developed by a community-based organization increased the number of individuals regularly testing for HIV, chlamydia, and gonorrhea, as well as their intention to test for HIV and other STIs [16–18].

Our study obtained data regarding the acceptability of using online media—specifically, a community-based audio drama titled *Hot Peach Tea*—to increase the awareness and uptake of PrEP among Asian-born MSM in Australia. This was compared with written information on a PAN website. The insights obtained will inform improvements to the audio drama and the trial process, contributing to the design and execution of future national clinical trials.

## METHOD

### Trial Design

This 1:1, open-label, 2-arm pilot study based on a stratified RCT evaluated the effectiveness, scale-up feasibility, and acceptability of the winning prototype, the *Hot Peach Tea* audio drama, from our 3-day designathon in April 2023 [20]. Our audio drama was further developed by the winning team, researchers, community members, and professional scriptwriters between July 2023 and March 2024. Community members provided feedback on the characters, contents, and audio during 3 focus groups and by email. This trial was conducted online in Australia between April and July 2024. Surveys were administered via Qualtrics provided by Monash University. The survey was developed by the research team and pilot tested among 5 community members to ensure clarity and comprehension. The link to the eligibility survey was disseminated to potential participants through the Melbourne Sexual Health Centre, Monash University Health Service, and local community organizations in Australia: Ending HIV Network, Australia and New Zealand Tongzhi Rainbow Alliance, Gay Asian Proud, Metro North Public Health Unit (Windsor, Queensland), Rapid Program, Queensland Positive People, and Ethnic Communities Council of Queensland. This study is reported according to the 2010 CONSORT guidelines. The trial stopped when we received 200 eligible responses.

### Study Populations

Our study included individuals who self-identified as gay, bisexual, queer, or other MSM; were born in southeast, east, or south Asian countries; were at least 18 years old at the time of recruitment; had no prior HIV diagnosis or positive HIV test result or had an unknown HIV status; had never used on-demand or daily HIV PrEP; had sufficient English to provide informed consent; and were willing to consent to the study. Exclusion criteria included individuals who self-identified as women, transgender men, or transgender women. Regarding sample size, with a total sample of 128 MSM (64 per arm), we would have 80% power to detect an increase in the intention-to-use PrEP scores from 28% to 52% (*P* < .05). For an assumed 8-week loss to follow-up of 30%, at least 184 MSM would need to be recruited (92 MSM per arm) to allow for a well-powered primary analysis. Further details on excluded responses and sample size calculation can be found in Supplementary Appendix 1.

### Ethics Approval

This study was registered at the Australian New Zealand Clinical Trials Registry (ACTRN12623001361695) and received ethics approval from the Alfred Ethics Committee (project 685/23).

### Intervention and Control

The intervention was an audio drama composed of 6 podcast-style episodes in English. The synopsis and main theme of each episode can be found in Supplementary Appendix 2 and Supplementary Table 1. The length of each episode is about 5 minutes, with a total duration of around 30 minutes. The script was written by Asian-born MSM living in Australia with the assistance of professional scriptwriters and then reviewed by the research team and community members.

In the control arm, participants received a link to PAN (PAN.org.au), a publicly available website. The PAN website was the common approach suggested by clinicians in Victoria to gain PrEP-related information, including how to initiate, purchase, and use PrEP in Australia with or without Medicare.

### Randomization

Participants were randomized into 2 arms via a randomizer integrated within Qualtrics, a web-based online management service. Simple randomization was performed within each stratum, without the use of predefined block sizes. Allocation concealment was ensured, as the service did not release the randomization code. Overall, 200 participants were randomly assigned to either the intervention arm (*Hot Peach Tea* audio drama) or the control arm (PAN website), with a 1:1 allocation per Qualtrics-generated randomization. Stratification was based on length of stay in Australia, categorizing participants as newly arrived MSM (≤4 years) and long-term stay MSM (>4 years) to ensure the balance in arrival time.

### Procedure

After completing electronic informed consent and confirming their eligibility, participants filled out a preintervention survey, including their demographic characteristics and contact information (ie, email address and phone number), as well as questionnaires assessing their knowledge of HIV and PrEP, attitudes toward PrEP, and intention to use PrEP. The questionnaires were adapted from Walsh's questionnaires [21]. The survey platform (Qualtrics) then randomized participants into the intervention arm (*Hot Peach Tea* audio drama) or the control arm (PAN website).

**Figure 1.**
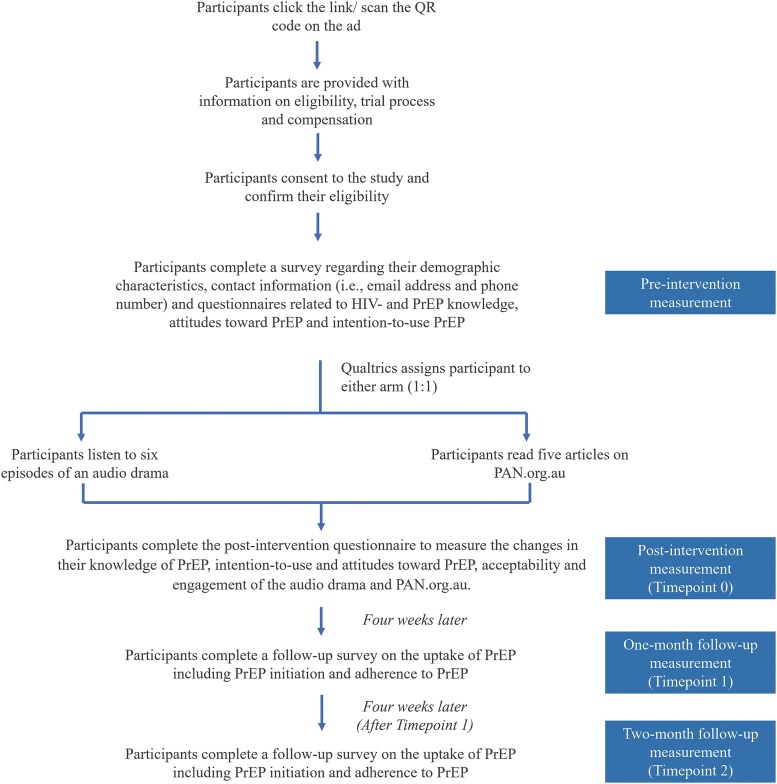
Trial implementation process. PAN, PrEP Access Now; PrEP, preexposure prophylaxis for HIV.

Participants in the intervention arm were directed to listen to 6 episodes of the audio drama, while participants in the control arm were directed to read the PAN website. We tracked the time that each participant spent listening to or reading the online media to assess engagement. Participants subsequently completed the postintervention questionnaire (time point 0) to measure the changes in their knowledge of PrEP, intention to use and attitudes toward PrEP, and satisfaction with the online media. At 1 and 2 months after the intervention (time points 1 and 2), participants received a follow-up email on the uptake of PrEP, including initiation and adherence. The survey asked participants whether they initiated PrEP in the past 4 weeks postintervention. If they initiated PrEP, we requested that they upload evidence, such as images of PrEP prescriptions or bottles, and we asked how they took PrEP. If participants did not initiate PrEP, we asked for their reasons. If no response was received, a follow-up email was sent weekly for 3 weeks (Figure 1).

E-gift vouchers of A$100 were offered as compensation for their time and to encourage sustained participation throughout the study. The use of incentives adhered to ethical guidelines and was designed to minimize attrition and maintain engagement, particularly considering the extended follow-up period.

### Outcomes

The outcomes are presented in Table 1, and the surveys can be found in Supplementary File 2. Our primary outcomes included acceptability (ie, satisfaction and engagement) of using online media to increase PrEP awareness (ie, attitudes) and uptake (ie, intention to use). Our primary outcomes were evaluated postintervention (time point 0). Our secondary outcomes included PrEP-related knowledge and PrEP initiation and adherence. Secondary outcomes were evaluated postintervention (time point 0) and at 1- and 2-month follow-ups (time points 1 and 2). PrEP-related knowledge was defined as changes in knowledge of PrEP postintervention. Further details on outcome definition and measurement can be found in Supplementary Appendix 3.

**Table 1.** Primary and Secondary Outcomes Measures and Time Points of Measurement

Outcome	Measure	Time Point 0: Postintervention	Time Point 1: Follow-up at 1 month	Time Point 2: Follow-up at 2 months
**Primary**				
Satisfaction	Participants’ satisfaction with intervention and control.	×		
Engagement	Percentage of participants who complete intervention and control.	×		
Intention to use PrEP	Changes in participants’ intention to use PrEP per a 3-item PrEP intention questionnaire by Walsh [21]	×		
Attitudes toward PrEP	Changes in participants' attitudes toward PrEP via a survey adapted from a 5-item PrEP attitudes questionnaire by Walsh [21]	×		
**Secondary**				
Change in knowledge of PrEP	Change in knowledge of PrEP per a survey adapted from Walsh's questionnaire [21]	×		
PrEP initiation after 1 month	Percentage of individuals who take further actions to obtain PrEP or initiate PrEP at 1 and 2 months after receiving the intervention or control.		×	
PrEP initiation after 2 months				×
Adherence to PrEP after 1 month	If participants initiate PrEP after receiving the intervention or control, we ask how they take PrEP (ie, daily or on demand), how many pills they have taken after they receive PrEP, and their reasons for discontinuing PrEP if they stop using it.		×	
Adherence to PrEP after 2 months				×

Blank cells indicate *not applicable*.

Abbreviation: PrEP, preexposure prophylaxis.

### Statistical Analysis

Differences in engagement in the online media and PrEP uptake between the arms were examined by χ^2^ tests. HIV-related knowledge scores, acceptability and engagement of the intervention, reasons for not initiating or discontinuing PrEP, and PrEP uptake and adherence were summarized with descriptive statistics. Changes in PrEP-related knowledge, intention to use PrEP, and attitude toward PrEP between the arms were compared by a nonparametric equality-of-medians test, with the median, IQR, range, and *P* value reported. Score calculation for PrEP-related knowledge, intention to use PrEP, and attitude toward PrEP was based on Walsh's study [21]. Intention-to-treat analysis was used where appropriate. All statistical analyses were conducted in STATA BE version 18.0 (StataCorp).

## RESULTS

Out of 1603 responses, 1403 were excluded. Further details on excluded responses can be found in Supplementary Appendix 1. Among 200 eligible responses, 96 were randomized into the control arm (PAN) and 104 in the intervention arm (*Hot Peach Tea* audio drama; Supplementary Figure 1). The median age was 29 years (IQR, 26–32; range, 20–44) for the PAN arm and 28 years (IQR, 25–31; range, 18–54) for the *Hot Peach Tea* arm. Most had been in Australia for at least 4 years (87% for the PAN arm and 77% for the *Hot Peach Tea* arm), were fully confident in their English level (76% and 65%), and had more than 1 sexual partner in the last 6 months (75% and 77% ). The demographic characteristics can be found in Table 2.

**Table 2.** Demographic Characteristics (N = 200)

	Control: PAN (n = 96)	Intervention: *Hot Peach Tea* (n = 104)
Age, y, median (IQR; range)	29 (26–32; 20–44)	28 (25–31; 18–54)
Self-identified as		
Gay or homosexual	89 (93)	90 (87)
Bisexual	6 (6)	7 (7)
Queer	1 (1)	5 (5)
Straight or heterosexual	0 (0)	2 (2)
Region of birth		
Southeast Asia	34 (35)	31 (30)
South Asia	16 (17)	16 (15)
Northeast Asia	46 (48)	57 (55)
Employment		
Full-time	56 (58)	48 (46)
Part-time	32 (33)	36 (35)
Student	2 (2)	8 (8)
Unemployed	6 (6)	11 (11)
Prefer not to answer	0 (0)	1 (1)
Medicare		
Yes	62 (65)	53 (51)
No	34 (35)	51 (49)
Length of stay in Australia		
>4 y	81 (87)	75 (77)
≤4 y	12 (13)	22 (23)
Level of English		
Fully confident	73 (76)	68 (65)
Moderately confident	50 (21)	26 (25)
Slightly confident	1 (1)	1 (1)
Somewhat confident	2 (2)	9 (9)
No. of sexual partners in the last 6 mo		
None	1 (1)	2 (2)
1	23 (24)	22 (21)
>1	72 (75)	80 (77)
Ever tested for HIV		
Yes	93 (97)	99 (95)
No	3 (3)	5 (5)

Data are presented as No. (%) unless noted otherwise.

Abbreviation: PAN, PrEP Access Now.

### Primary Outcomes

#### Satisfaction

**Figure 2.**
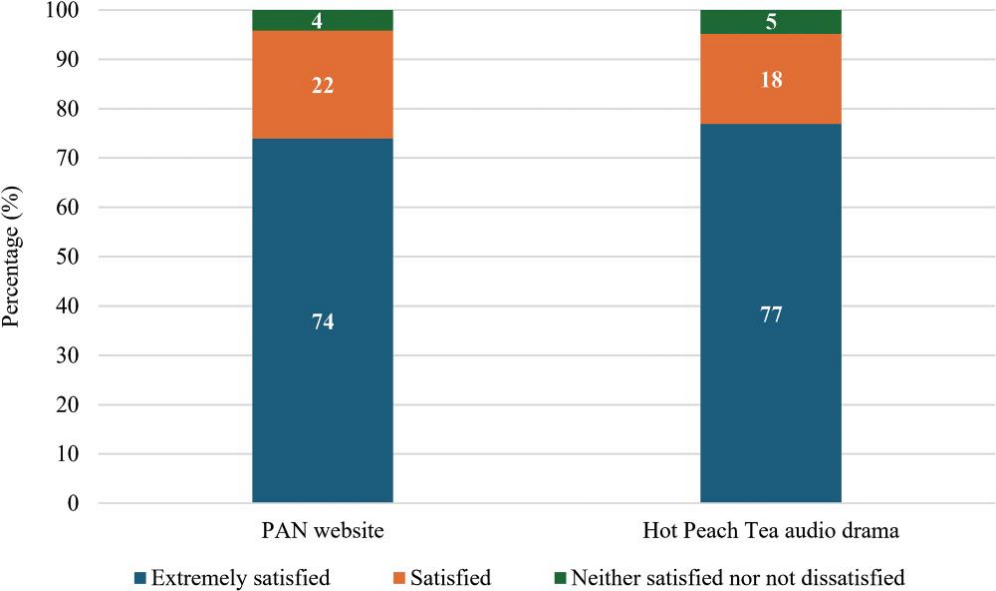
Participants’ satisfaction with the *Hot Peach Tea* audio drama and the PAN website. PAN, PrEP Access Now.

In the PAN arm, 74% (71/96) of participants were extremely satisfied with their online media postintervention (time point 0), as compared with 77% (80/104) in the *Hot Peach Tea* arm (Figure 2).

#### Engagement

**Figure 3.**
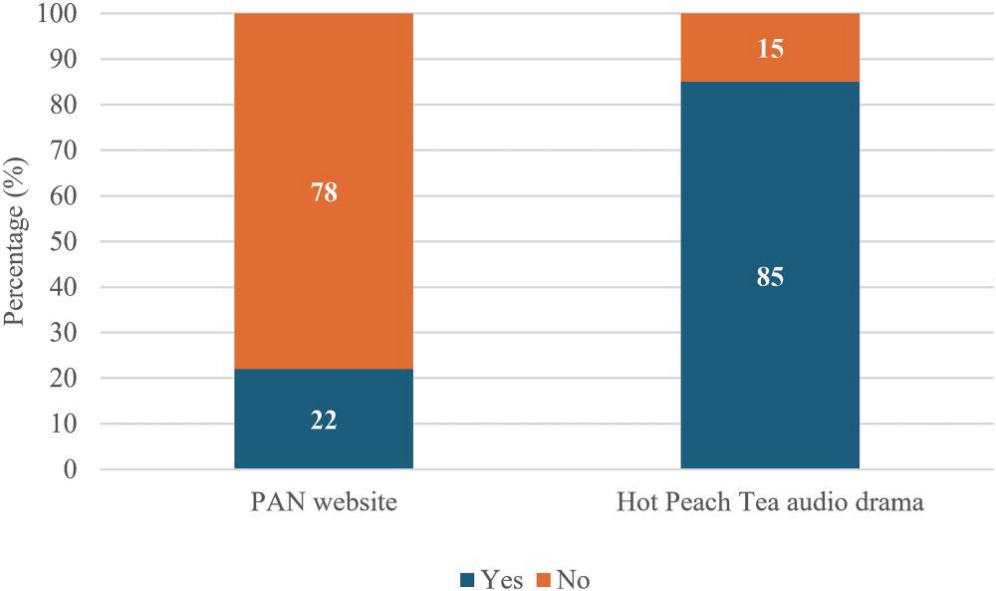
Number of participants engaged in the *Hot Peach Tea* audio drama and the PAN website. PAN, PrEP Access Now.

There was a higher percentage of participants engaged in the *Hot Peach Tea* audio drama (85%, 88/104) as compared with the PAN website (22%, 21/96, *P* < .001; Figure 3). The details of engagement levels for each arm can be found in Supplementary Figure 2.

#### Changes in Attitude Toward PrEP

The median score for attitude toward PrEP significantly improved from 33 (IQR, 30–36) preintervention to 38 (32–42) postintervention (time point 0) among participants in the PAN arm (*P* < .001) and from 33 (30–36) to 40 (36–46) in the *Hot Peach Tea* arm (*P* < .001). The positive change in attitude toward PrEP increased in both groups postintervention but was higher in the *Hot Peach Tea* arm (*P* = .023; Table 3).

**Table 3.** Comparison of Outcomes Measured at Postintervention and 1- and 2-Month Follow-up (Time Points 0, 1, and 2)

Outcome	Control: PAN Website	Intervention: *Hot Peach Tea* Audio Drama	*P* Value^a^
Intention to use PrEP (range, 3–12)			.056
Preintervention	9 (7–10; 3–12)	9 (8–10; 3–12)	
Postintervention	11 (10–12; 4–12)	12 (10–12; 6–12)	
Changes in intention to use PrEP	2 (0–3; −3, 9)	2 (0–3; −2, 9)	
Attitude toward PrEP (range, 10–50)			.023
Preintervention	33 (30–36; 26–42)	33 (30–36; 21–48)	
Postintervention	38 (32–42; 26–50)	40 (36–46; 13–50)	
Changes in intention to use PrEP	4 (0–7; −13, 19)	7 (3–12.5; −11, 20)	
PrEP-related knowledge (range, 0–10)			.366
Preintervention	1 (0–1; 0–4)	0 (0–1; 0–6)	
Postintervention	1 (0–2; 0–8)	1 (1–1; 0–7)	
Changes in intention to use PrEP	0 (0–1; −2, 5)	0 (0–1; −2, 5)	

Data are presented as median (IQR; range).

Abbreviation: PrEP, preexposure prophylaxis.

^a^ *P* value measures the difference between the change in outcomes (pre- vs postintervention) between the groups.

#### Changes in Intention to Use PrEP

The median score for intention to use PrEP significantly increased from 9 (IQR, 7–10) preintervention to 11 (10–12) postintervention (time point 0) among participants in the PAN arm (*P* < .001) and from 9 (8–10) to 12 (10–12) in the *Hot Peach Tea* audio drama arm (*P* < .001). A median change in intention to use PrEP between pre- and postintervention was 2 (IQR, 0–3) in the PAN arm and 2 (0–3) in the *Hot Peach Tea* arm. However, there was no significant difference between the arms (*P* = .056; Table 3).

### Secondary Outcomes

#### PrEP Initiation at 1 and 2 Months After Receiving the Online Media

At the 1-month follow-up (time point 1), 50% (48/96) of participants in the PAN arm took action to initiate PrEP, and 14% (13/96) started PrEP. The top 3 reasons for not initiating PrEP included concern about side effects (n = 20), low self-perceived risk of HIV infection (n = 16), and preference for condoms (n = 15). Meanwhile, in the *Hot Peach Tea* arm, 49% (51/104) took action to initiate PrEP, and 9% (9/104) initiated PrEP. The top 3 reasons for not initiating PrEP included the inconvenience of using PrEP (n = 27), privacy concerns (n = 24), and a preference for using condoms (n = 15). There was no significant difference in PrEP initiation at the 1-month follow-up between the arms (14% in the PAN arm vs 9% in the *Hot Peach Tea* arm).

**Figure 4.**
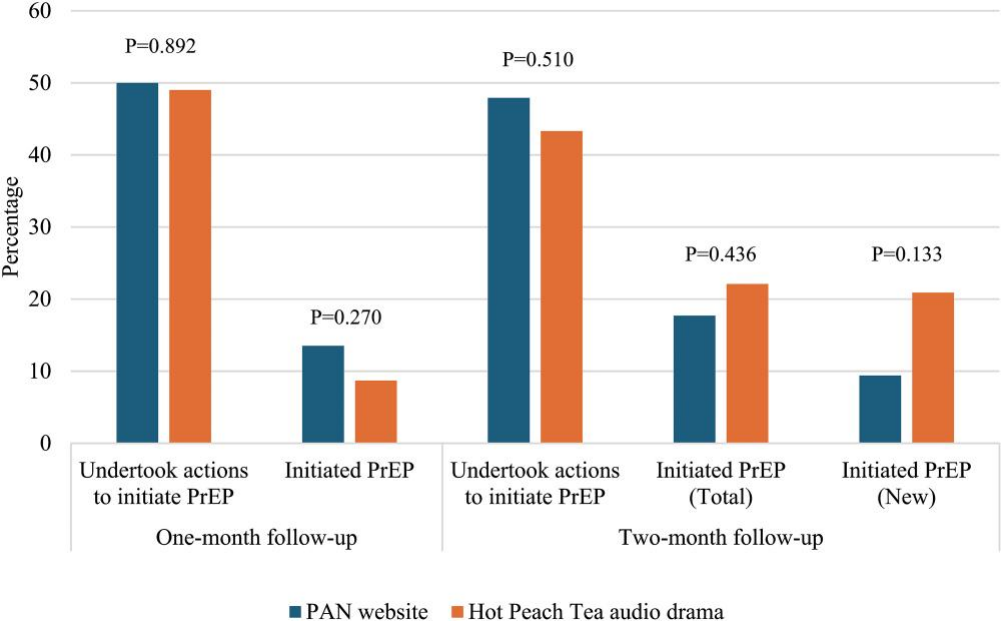
The uptake of PrEP at 1- and 2-month follow-ups. The figure shows percentages of the uptake of PrEP at 1 and 2 months after time point 0. *P* < .05 indicates a statistically significant difference in uptake between the arms. PAN, PrEP Access Now; PrEP, preexposure prophylaxis.

At the 2-month follow-up (time point 2), 48% (46/96) of participants in the PAN arm took action to initiate PrEP, and 18% initiated PrEP (17/96). Of the 17 who initiated PrEP, 9 were new initiations. The top 3 reasons for not initiating PrEP included concern about side effects (n = 19), the inconvenience of using PrEP (n = 14), and preference for condoms (n = 14). Meanwhile, in the *Hot Peach Tea* arm, 43% (45/104) took action to initiate PrEP, and 22% (23/104) initiated PrEP. Of the 23 who initiated PrEP, 18 were new initiations. The top 3 reasons for not initiating PrEP included inconvenience to use PrEP (n = 17), privacy concerns (n = 15), and concern about side effects (n = 15). No significant difference in PrEP initiation between the arms was observed at the 2-month follow-up (18% in the PAN arm vs 22% in the *Hot Peach Tea* arm; Figure 4, Supplementary Table 2).

#### Adherence to PrEP at 1 and 2 Months After Receiving the Online Media

At the 1-month follow-up (time point 1), only 1 (1%) participant in the PAN arm discontinued PrEP after initiation and 2 in the *Hot Peach Tea* arm (2%). Reasons for discontinuation included challenges in consistent PrEP adherence and entering a committed relationship. In the 2-month follow-up survey (time point 2), 6 (6%) participants discontinued PrEP in the PAN arm and 4 (4%) in the *Hot Peach Tea* arm. Cited reasons included side effects, financial barriers, entering into a committed relationship, and difficulties in consistent PrEP adherence.

The median number of daily oral PrEP pills taken in the 1-month follow-up survey (time point 1) was 16 (IQR, 10–2; range, 8–27) in the PAN arm and 20 (16–26; 8–30) in the *Hot Peach Tea* arm. In the 2-month follow-up survey (time point 2), the median number of pills taken in month 2 was 21 (IQR, 17–22) in the PAN arm and 19 (12–25) in the *Hot Peach Tea* arm.

#### Changes in PrEP-Related Knowledge

The median change in PrEP knowledge score between pre- and postintervention (time point 0) was 1 (IQR, 0–1; range, 0–4) in the PAN arm and 0 (0–1; 0–6) in the *Hot Peach Tea* arm. There was no significant difference in the changes in PrEP knowledge score between the arms (*P* = .056; Table 3).

## DISCUSSION

This study was an open-label, 2-arm pilot study based on a stratified RCT measuring the acceptability of using online media to increase the awareness and uptake of PrEP through a community-based audio drama called *Hot Peach Tea*, as compared with written information on the PAN website, among Asian-born MSM in Australia. Our findings demonstrated that most participants in both arms expressed extreme satisfaction with the online media that they received, and they were more likely to use PrEP after receiving the online media. Both online media increased PrEP initiation among Asian-born MSM with low knowledge of HIV and PrEP. Despite no significant difference in uptake of PrEP and changes in knowledge related to PrEP between the arms, more participants were engaged in the *Hot Peach Tea* audio drama as compared with the PAN website. Furthermore, participants in the *Hot Peach Tea* arm had a greater increase in the score of having a positive attitude toward PrEP. Our study findings highlight the importance of PrEP interventions tailored to Asian-born MSM to engage and positively influence their attitudes toward PrEP. The combination of different interventions and strengthening linkage to care has the potential to increase the uptake of PrEP among this population.

The community-codesigned *Hot Peach Tea* audio drama demonstrated greater potential in engaging Asian-born MSM and positively influencing their attitudes toward PrEP as compared with control. Previous qualitative interviews reported that Asian-born MSM perceived PrEP as not applicable for them because Asians were not perceived as “highly sexually active individuals” [11, 12]. Our findings showed that the *Hot Peach Tea* audio drama addressed this self-perception related to PrEP users, as evidenced by the observed improvement in attitudes toward PrEP. By narrating the personal journey of a newly arrived Asian-born man in the MSM community who initiated PrEP to protect himself from acquiring HIV, the audio drama normalized PrEP use without associating it with heightened sexual activity. Instead, it presented PrEP as a mainstream pragmatic choice for HIV prevention, aligning with the context of sexual life in Australia [22]. As observed from greater engagement and a positive score increase in attitude toward PrEP in our study, this community-codesigned narrative approach may offer unique advantages over more traditional educational interventions. Specifically, traditional educational interventions can be too formal or feel patronizing to migrant groups, but storytelling makes the educational content more relevant and creates room for self-reflection [23]. Additionally, the format of the audio drama could foster behavior change over time as participants internalized the health message. This is supported by the higher absolute number of PrEP initiations at the 2-month follow-up in the *Hot Peach Tea* arm, which was twice that of the PAN website arm.

Our community-codesigned audio drama *Hot Peach Tea* has the potential to increase the PrEP initiation rate, but addressing the gap in linkage to care is essential to achieving this. Our findings showed no significant difference in the percentage of PrEP initiation between the arms. However, we hypothesize that this observation occurred because participants in the PAN website arm were directly provided with a list of PrEP providers and could schedule an appointment during the trial in addition to receiving free PrEP via a coupon system. Meanwhile, participants in the *Hot Peach Tea* arm, while highly engaged with the audio drama, lacked a direct linkage to care after completing the audio drama. The process of initiating PrEP in Australia can be complicated and overwhelming, particularly for individuals from overseas. It usually involves making an appointment with a doctor, visiting a clinic or sexual health center, undertaking an HIV test, waiting for the result (which can take up to a week), obtaining a PrEP prescription, and purchasing PrEP [24]. The PAN website facilitated this process by linking individuals to each step, streamlining their PrEP initiation process. Yet, participants in the *Hot Peach Tea* arm had to navigate these steps independently, which might have been overwhelming, and without Medicare they would face additional costs. This is supported by the most reported reason for not initiating PrEP in the *Hot Peach Tea* arm during the 1-month follow-up, which was the perceived inconvenience to use PrEP. Offering the *Hot Peach Tea* audio drama with the PAN website resources to link to care could bridge this gap. The drama can serve as an engaging entry point, helping to reshape perceptions and reduce stigma surrounding PrEP use among Asian-born MSM. Simultaneously, the PAN website could provide a seamless transition to health care, linking participants to PrEP providers and offering free PrEP to help initiate their prevention journey. Our future trial should include access to online media and access to only the PAN website to better investigate the effectiveness of the *Hot Peach Tea* audio drama, since these 2 online media could be delivered together. Another approach could be integrating the *Hot Peach Tea* audio drama into community-led clinics or waiting room environments at sexual health centers, which could enhance PrEP uptake. This approach has shown promise in a controlled trial in the United States, where a theory-based 23-minute video portraying couples overcoming barriers to safer sexual behaviors was implemented in the waiting rooms of 3 STI clinics over a 21-month period. A nearly 10% reduction in new STI incidence was observed during the implementation period [25]. By adopting this approach, *Hot Peach Tea* could create an environment for proactive HIV prevention during clinic visits.

Effective learning is often a gradual process that requires repeated exposure to information over time [26]. Our trial showed a low level of PrEP knowledge among participants in both arms, even after the interventions. Delivering a large volume of information all at once, especially in a participant's second language, may contribute to limited improvements in knowledge scores. This highlights the importance of reinforcing key messages consistently over time to enhance understanding. Future trials should incorporate more interactive elements into the intervention to improve PrEP knowledge and initiation. This could include follow-up educational sessions or supplementary resources, allowing participants to revisit and solidify the information at their own pace. Additionally, delivering content through their first language, video, or infographic format may support retention, particularly for individuals with varying levels of language proficiency. Our trial findings provide valuable insights to improve future community-based interventions for Asian-born MSM in Australia.

We acknowledge several limitations of this trial. First, we did not collect data on when participants initiated PrEP, which limited our ability to fully assess adherence among those who reported starting daily PrEP and were unable to provide the denominator for PrEP pills taken. Second, we experienced a loss in follow-up of approximately one-fourth of the participants during the 2-month follow-up survey. However, we mitigated the risk of overestimating the effectiveness of the intervention by conducting an intention-to-treat analysis. Third, our trial relied on self-reported data for PrEP initiation, which may introduce bias. To enhance the reliability of the data, we asked participants to upload evidence, such as images of doctor's appointment confirmations or PrEP prescriptions. Fourth, we did not collect data on other PrEP-related interventions or education that participants might receive during the trial, and most participants, who were long-term settlers, may know access points to HIV prevention. Yet, we collected data on whether they searched for PrEP information. Our analyses also evaluated changes in intention to use PrEP, attitudes toward PrEP, and PrEP-related knowledge between the groups to provide a more comprehensive assessment of the intervention's impact.

In conclusion, the *Hot Peach Tea* audio drama, codesigned by the local community, demonstrated promise in engaging Asian-born MSM to learn about PrEP and positively influence their attitude toward PrEP use. Strategies to effectively link individuals to care after listening to the audio drama are needed to increase the uptake of PrEP. Future trials should consider incorporating follow-up educational sessions and additional resources on PrEP and explore the use of drama in video formats in different languages to support the learning process for this population.

## Supplementary Material

ofaf321_Supplementary_Data

## Supplementary Data

Supplementary materials are available at *Open Forum Infectious Diseases* online. Consisting of data provided by the authors to benefit the reader, the posted materials are not copyedited and are the sole responsibility of the authors, so questions or comments should be addressed to the corresponding author.

## Notes

***Acknowledgments.*** We acknowledge, in alphabetical order, the content creator team (ie, the winning team from the designathon), including Alexander Luciano Hudson (Hello@lucianoart.ist), Riley J. Siu (rileyjsiu@icloud.com), Satrio Nindyo Istiko, and Shushen Feng, for their development of the *Hot Peach Tea* audio drama. We also extend our gratitude to Rae Choi (contact@rachelchoi.com) and Joshua Tate for their professional scriptwriting services, and Rudy Bilani for his professional soundtrack composition service. We thank Shushen Feng for voicing Xiaolin, James Tapa for Sunny, James Seow for Derek, Rudy Bilani for James, Phyu Mon Latt for the doctor, and Nyi Nyi Soe and Riley for extra characters.

We are grateful for the support from the community for its contributions to the development of the audio drama and the survey used in this trial: Duy Tran (ACON, New South Wales 2000), Cedric Yin-Cheng (Australian and New Zealand Rainbow Alliance, New South Wales 2150), Joean Yun (Victoria), Maxi Xie (Australian and New Zealand Rainbow Alliance), Nutthanon Ngamchaipisit (Western Australia AIDS Council, Perth 6005), and Tim Wark (ACON, New South Wales 2000).

We further acknowledge the clients and staff at the Melbourne Sexual Health Centre, James Seow (Gay Asian Proud, Victoria), Joseph Debattista (Metro North Public Health Unit), Judith Dean (University of Queensland), Luke Coffey (Rapid Program, Queensland Positive People), and Zhihong Gu (Ethnic Communities Council of Queensland) for their support in recruiting trial participants.

Finally, we express our deepest appreciation to the participants of the crowdsourcing open call and designathon, whose contributions led to *Hot Peach Tea* emerging as a winning solution.

***Author contributions.*** J. J. O. and W. T. conceived the idea for the manuscript. S. N. I., W. T., J. T., and J. W. contributed to the development of the intervention. W. T., J. J. O., C. K. F., T. R. P., E. P. F. C., and L. M. contributed to the methodology of this study. J. J. O., C. K. F., T. R. P., E. P. F. C., and L. Z. contributed to the supervision of this study. W. T. designed the survey. W. T., J. J. O., S. N. I., and J. W. contributed to the design and delivery of the survey. W. T. recruited participants, collected the data, conducted the analysis, wrote the first draft of the manuscript, and revised and finalized the manuscript. E. P. F. C. and J. J. O. provided statistical advice. All authors contributed to the manuscript and approved the final version for submission.

***Financial support.*** This work was supported by the National Health and Medical Research Council (GNT1193955 to J. J. O. and GNT1172873 and GNT2033299 to E. P. F. C.).
