# Supplementary material for: Using Online Media to Increase the Awareness and Uptake of Preexposure Prophylaxis for HIV Among Asian-Born Men Who Have Sex With Men Living in Australia: An Open-Label Randomized Controlled Trial
Source: Open Forum Infect Dis. 2025 Jul 23;12(7):ofaf321. doi: 10.1093/ofid/ofaf321 (PMC12284882; doi:10.1093/ofid/ofaf321)
Supplement: ofaf321_Supplementary_Data [file ofaf321_supplementary_data.zip › Supplementary 1 V0.3 25January13.docx]

**Appendix 1.**

**Excluded responses**

We manually excluded responses from participants who did not pass the verification process (i.e. did not respond to our verification email, failed to answer the verification questions, or attempted the survey multiple times using the same email address and phone number). We also excluded responses that appeared to be from bots (i.e., answered the honeypot question (hidden questions that can only be answered by bots), used overseas phone numbers, or submitted responses from outside Australia).


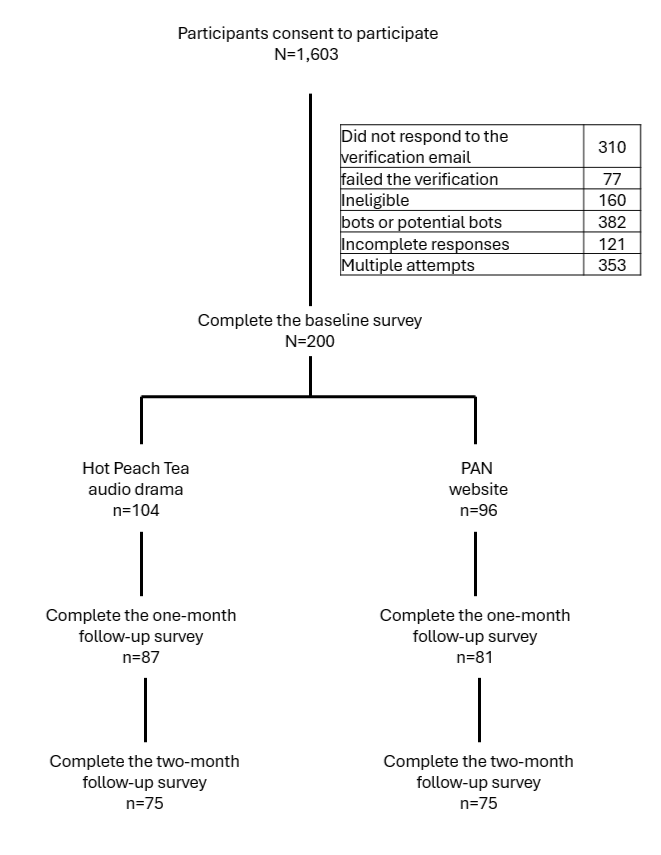


**Figure S1. Inclusion and exclusion criteria**

**Sample size**

In a previous study, compared to treatment-as-usual, MSM who received a behavioural intervention to promote PrEP use were more likely to use PrEP (52.3% (intervention) versus 27.9% (treatment-as-usual); P=0.005).^25^ With a total sample size of 128 MSM (64 per arm), we have 80% power to detect an increase in the intention-to-use PrEP scores from 28% to 52% (P<0.05). Assuming an 8-week loss to follow-up of 30%, at least 184 MSM need to be recruited (92 MSM per arm) to allow for a well-powered primary analysis. Additionally, according to rules of thumb, 60-100 participants per group is sufficient for binary outcomes in a pilot study.^26^

**Appendix 2.**

**The synopsis of the Hot Peach Tea audio drama**

The narrative featured three queer Asian-born MSM main characters, including a newly-arrived MSM who was a naïve PrEP user (Xiaolin, a 22-year-old gay man, new to the gay scene), an experienced PrEP user who was vulnerable to HIV infection (Sunny, a 29-year-old queer man) and another experienced PrEP user who was at low risk of HIV infection (Derek, a 42-year-old community older and mentor). Designed by community members, the audio drama tackled common misconceptions and barriers to PrEP implementation. It showed the self-growth of the newly-arrived MSM, to increase relatability to Asian-born MSM newcomers to Australia while delivering health messages to the listeners in a fun and easy-to-follow manner. Examples of the messages were ‘enjoy safe sex, get PrEP’ and ‘PrEP is for everyone, being on PrEP doesn’t mean saying goodbye to your culture’.

**Table S1. Contents of each episode of the *Hot Peach Tea* audio drama**

| **Episode** | **Title** | **Health Message** | **Content** | **Addressing barriers to access PrEP in Australia** |
| --- | --- | --- | --- | --- |
| 1 | He asked me if I was on PrEP… I said I douched | Enjoy safe sex, get PrEP | Xiaolin, the gay baby, failed his first attempt to hook up in Australia because he didn’t know about PrEP. | Do not know what PrEP is |
| 2 | To PrEP or not to PrEP | PrEP is for everyone, being on PrEP doesn’t mean saying goodbye to your culture | Xiaolin researched PrEP and discussed his barriers to accessing PrEP with his friends, Sunny and Derek. | Debunk myths around PrEP   1. High cost without Medicare 2. Slut-shaming towards PrEP users* 3. PrEP interaction with Chinese herbal medicine* 4. Distrust of Western medicine* 5. Do not want the family to know* |
| 3 | How would you like PrEP | Choose how you want to PrEP, choose how you want to have fun | Derek took Xiaolin to a clinic to get PrEP. | 1. Do not know how to access PrEP 2. Do not want the family to know* |
| 4 | Sex after PrEP | I’m happier now I’m PrEPared | Xiaolin had a happier and safer dating life after taking PrEP. Xiaolin encountered the first guy he hooked up with. | PrEP side effects |
| 5 | No respect, No sex | PrEP is great for sex, but great sex has to be consensual | Xiaolin was concerned that the guy took a condom off during a sexual encounter without his consent. But it was just an accident. Xiaolin felt safe from HIV because he was PrEP. | 1. PrEP effectiveness 2. do not talk about sex or HIV* |
| 6 | In the end, community matters | A good social life equals a good sexual life | Xiaolin reflected on his sexual life journey in Australia and wanted to contribute to the community. | 1. Pill fatigue 2. Stigma around being a PrEP user |

PrEP=pre-exposure prophylaxis

*Cultural barriers

**Appendix 3.**

**Primary outcome definition and measurement**

Our primary outcomes included acceptability (i.e., satisfaction and engagement) of using online media to increase awareness (i.e., attitudes towards PrEP) and uptake of PrEP (i.e., intention-to-use PrEP). Our primary outcomes were evaluated post-intervention (Timepoint 0). Satisfaction was defined as participants’ satisfaction with intervention or control. Engagement was defined as the percentage of participants who completed the intervention and control. Awareness was measured through attitudes towards PrEP.^24^ Attitudes were defined as changes in participants’ attitudes toward PrEP post-intervention. Uptake of PrEP was measured through intention-to-use PrEP. Intention-to-use PrEP was defined as changes in participants’ intention to use PrEP post-intervention.

We evaluated participants’ satisfaction with the online media they received using a 5-point Likert scale and their willingness to recommend the online media to peers. Engagement was evaluated by determining the percentage of participants who completed the online media, defined as watching episodes one through six of the audio drama for between 15 (if they increased the speed of the audio drama or skipped the intro and ending) and 60 minutes or reading the five articles from the *PAN* website for 10 to 20 minutes. Changes in participants’ intention to use PrEP and attitudes toward PrEP were assessed using questionnaires adapted from Walsh’s questionnaires.^24^ For example, participants responded to this statement ‘During the next three months, I will talk to a healthcare provider about PrEP’. Responses were on a scale from ‘definitely will not do’ (1) to ‘definitely will do’ (4), with higher scores indicating greater intention to use PrEP. Our primary outcomes can be found in Table 1.

**Secondary outcome definitions and measurement**

Our secondary outcomes included knowledge and PrEP initiation and adherence. Secondary outcomes were evaluated post-intervention (Time point 0) and at one- (Time point 1) and two-month follow-ups (Time point 2). PrEP initiation was defined as the percentage of individuals who took further actions to obtain PrEP or initiated PrEP at one- and two months post-intervention. Adherence to PrEP was defined as how participants took PrEP (i.e., daily or on-demand), how many pills they took and their reasons for discontinuing PrEP if applicable.

We measured PrEP initiation by calculating the percentage of individuals who reported taking further actions to initiate PrEP, such as researching more about PrEP, speaking to a doctor or nurse about PrEP, or initiating PrEP within one- and two-month post-intervention. Participants who did not start PrEP were asked to provide reasons for their decision in the survey. We assessed adherence to PrEP by inquiring how participants used PrEP (i.e., daily or on-demand), the number of pills taken and their reasons for discontinuing PrEP if applicable. We evaluated changes in knowledge of PrEP using a questionnaire adapted from the 13-item PrEP knowledge questionnaire by Walsh.^24^ Our secondary outcomes can be found in Table 1.

**Figure S2. Engagement levels between the PAN and Hot Peach Tea arms**

Fully engaged = Completing six episodes of the Hot Peach Tea audio drama between 15 and 60 minutes or five articles on the PAN website between 10 and 20 minutes

Partly engaged = Completing six episodes of the Hot Peach Tea audio drama between 8 and 14.9 minutes or five articles on the PAN website between 5 and 9.9 minutes

Not engaged = Completing six episodes of the Hot Peach Tea audio drama in less than 8 minutes or five articles on the PAN website in less than 5 minutes

**Table S2. Reasons for not initiating PrEP at one- and two-month follow-ups**

|  | **One-month follow-up** | | **Two-month follow-up** | |
| --- | --- | --- | --- | --- |
| **Reason for not initiating PrEP** | ***PAN* website (control)**  **n (%)** | ***Hot Peach Tea* audio drama (Intervention)**  **n (%)** | ***PAN* website (control)**  **n (%)** | ***Hot Peach Tea* audio drama**  **(Intervention)**  **n (%)** |
| Concern about PrEP side effects | 20 (21%) | 13 (13%) | 19 (20%) | 15 (14%) |
| Concern about drug interaction | 12 (13%) | 11 (11%) | 9 (9%) | 8 (8%) |
| Low self-perceived risk of HIV infection | 16 (17%) | 6 (6%) | 10 (10%) | 7 (7%) |
| Concern about privacy | 10 (10%) | 24 (23%) | 12 (13%) | 15 (14%) |
| Challenges in navigating the process of accessing PrEP | 3 (3%) | 11 (11%) | 2 (2%) | 2 (2%) |
| Preference for condoms | 15 (16%) | 15 (14%) | 14 (15%) | 11 (11%) |
| Concern about parents finding out | 7 (7%) | 6 (6%) | 9 (9%) | 8 (8%) |
| Inconvenience to use PrEP | 11 (11%) | 27 (26%) | 14 (15%) | 17 (16%) |
| High price of PrEP | 7 (7%) | 5 (5%) | 5 (5%) | 6 (6%) |
| Unaffordability | 6 (6%) | 5 (5%) | 5 (5%) | 3 (3%) |
| Lack of knowledge about PrEP | 6 (6%) | 3 (3%) | 0 (0%) | 1 (1%) |
