# Supplementary material for: Using Online Media to Increase the Awareness and Uptake of Preexposure Prophylaxis for HIV Among Asian-Born Men Who Have Sex With Men Living in Australia: An Open-Label Randomized Controlled Trial
Source: Open Forum Infect Dis. 2025 Jul 23;12(7):ofaf321. doi: 10.1093/ofid/ofaf321 (PMC12284882; doi:10.1093/ofid/ofaf321)
Supplement: ofaf321_Supplementary_Data [file ofaf321_supplementary_data.zip › Supplementary 2.pdf]

Recapcha

Before you proceed to the survey, please complete the captcha below.

☐ I'm not a robot
reCAPTCHA
Privacy - Terms

Introduction

How did you hear about this survey?

- ACON
- Melbourne Sexual Health Centre (MSHC)
- ANTRA
- Gay Asian Proud
- Better Health Network
- Ethnic Communities Council Queensland

- QLD Council for LGBT health
- Queensland Positive People
- QHealth
- Other (Please specify) ________

Are you a real person?

- Yes

Which of the following best describes your gender?

- Man
- Woman
- Non-binary
- Genderqueer/ Gender non-confirming
- Other (Please specify) ________

What sex were you assigned at birth (Meaning on your birth certificate)?

- Male
- Female
- Other (Please specify) ________

Do you identify yourself as:

- Gay/ Homosexual
- Straight/ Heterosexual
- Bisexual
- Queer
- Other (Please specify) ____

To whom are you sexually attracted?

- Men only
- Mainly men, some women
- Men and women
- Mainly women, some men
- Women only
- Other (Please specify)

You are not eligible to participate in this survey

How old are you?

____

What is your country of birth?

[ ]

Have you been diagnosed with HIV?

- ○ No
- ○ Yes
- ○ I have not had a HIV test before.

Before you were involved in this study, had you heard the term **PrEP** before?

- ○ Yes I have
- ○ No I have not

Have you ever taken PrEP?

- ○ Yes, I am currently taking PrEP
- ○ Yes I took PrEP more than 12 months ago
- ○ Yes I took PrEP within the last 12 months
- ○ No I have not

You are eligible to participate in this trial.

Please enter your email address and phone number on the next page.
Rest assured, we will only use your phone number for the purpose of **verifying before issuing the e-voucher, as we want to** ensure the integrity of our data and prevent fraudulent responses.
We will only send emails about our **follow up surveys** and **updates on the trial** to you.
No other information will be sent to your phone number, and your privacy will be strictly maintained. Your email address and phone number will not be shared with other projects or organisations.

You are not eligible to participate in this survey

Please enter your email address below (For example trial@gmail.com)

Please enter your Australian phone number below (For example 0412345678)

Before we start, we would like to ask questions about you.
Your answers will be private

In the past 6 months, in which Australian state have you spent the most time?

What is the highest level of education that you have completed?

- Less than high school
- High school graduate
- Bachelor degree
- Master degree
- Doctorate

What is your current employment status?

- Employed full time
- Employed part time
- Unemployed looking for work
- Unemployed not looking for work
- Retired
- Student
- Prefer not to answer

For how long have you lived in Australia?

Do you have an Australian Medicare card?

- Yes
- No

In general, how confident are you to speak English without preparation in advance?

- Fully confident
- Moderately confident
- Somewhat confident
- Slightly confident
- Not at all confident

How many male sexual partners did you have in the past 6 months?

- No sexual partner
- 1 sexual partner
- 2-3 sexual partners
- more than 3 sexual partners

How many female sexual partners did you have in the past 6 months?

- No sexual partner
- 1 sexual partner

- 2-3 sexual partner
- more than 3 sexual partner

Thank you for providing us with your information. The next section will be questions about PrEP and HIV.

Please complete this section without further researching the answer, as we would like to know your base knowledge on HIV and PrEP.

**Please note: you have the flexibility to return and finish this survey within the next 2 weeks by simply reopening the same link in the same browser.*

For each of the following questions, please choose "True", "False", or "Don’t Know" . If you do not know, please do not guess; instead, please select "Don’t Know".

	True	False	Don't know
I have heard about HIV and / or AIDS.	○	○	○
A HIV test is done whenever someone has a blood test in Australia.	○	○	○
It is safe to have sex without using HIV prevention methods (e.g., condoms or PrEP) with someone who has UNDETECTABLE HIV viral load.	○	○	○
There is medication available for people living with HIV so they can live a normal life.	○	○	○

	True	False	Don't know
There are medicines that people can take BEFORE- or AFTER SEX to protect themselves against HIV.	○	○	○

For each of the following questions, please choose True (T), False (F), or Don’t Know (DK). If you do not know, please do not guess; instead, please choose Don’t Know.

	True	False	Don't know
PrEP is a pill you can take after sex to reduce your risk of becoming infected with HIV.	○	○	○
There may be some herbal medicine that can reduce the effectiveness of PrEP in preventing HIV.	○	○	○
PrEP can be used to prevent sexually transmitted infections like gonorrhea, chlamydia, syphilis, and herpes.	○	○	○
PrEP can be taken by people who already have HIV.	○	○	○
If you start taking PrEP, you will have to take it for the rest of your life.	○	○	○
You need insurance or Medicare to access PrEP in Australia.	○	○	○
In Australia, I can get PrEP at a pharmacy without seeing a doctor.	○	○	○
You must take a HIV test every 3 months while taking PrEP in Australia.	○	○	○
There are many serious side effects of taking PrEP.	○	○	○
Only daily PrEP can lower the risk of getting HIV from sex.	○	○	○

How likely are you to do the following in the next 3 months?

	Definitely Will Not Do	Probably Will Not Do	Probably will do	Definitely will do
I will talk to a health care provider about PrEP	○	○	○	○
I will seek out more information about PrEP.	○	○	○	○
I will get a prescription for PrEP.	○	○	○	○

Do you agree or disagree with the following statement?

	Strongly disagree	Somewhat disagree	Neither agree nor disagree	Somewhat agree	Strongly agree
PrEP is effective at preventing HIV.	○	○	○	○	○
People who take PrEP are responsible.	○	○	○	○	○
Taking PrEP is safe.	○	○	○	○	○
It would be no trouble to take PrEP every day.	○	○	○	○	○
The government makes certain that drugs like PrEP are safe.	○	○	○	○	○

	Strongly disagree	Somewhat disagree	Neither agree nor disagree	Somewhat agree	Strongly agree
You need Australian citizenship or permanent residency to access PrEP in Australia.	○	○	○	○	○
PrEP is expensive for people without Medicare.	○	○	○	○	○
PrEP cannot be taken with any herbal medicine.	○	○	○	○	○
PrEP is for only highly sexually active people.	○	○	○	○	○
Being on PrEP is stigmatising.	○	○	○	○	○

Could you please share why you have not used PrEP?

- ☐ I don't know enough about PrEP
- ☐ I can afford to use PrEP but I don't want to pay the high price
- ☐ I cannot afford PrEP even though I want to use it.
- ☐ I am worried about the side effects of PrEP
- ☐ I am worried about PrEP interacting with other medications of herbal medicines
- ☐ I don't think I need PrEP (such as being single or in a committed relationship).
- ☐ I don't know how to access PrEP in Australia.
- ☐ I don't want other people to know that I'm on PrEP.
- ☐ I don't want to use my health insurance in case my parents find out I'm using PrEP.
- ☐ I prefer to just use condoms to protect myself against HIV.

- [ ] I find it too inconvenient to use PrEP.
- [ ] Other (please specify) ________

Halfway Through! You're Almost There to Receive AU$50!

Thank you for taking the time to answer questions about PrEP and HIV. You're making excellent progress. In the next section, you'll have access to online media that provides valuable information about PrEP.

After Completing the Intervention! You'll move on to the FINAL part of the survey, the post-intervention questionnaire related to PrEP and the online media.

**Please note: you have the flexibility to return and finish this survey within the next 2 weeks by simply reopening the same link in the same browser.*

Arm 1

In this section, you will learn about PrEP from **PAN.org.au**.

Please complete all the reading as listed below
1. Starting PrEP
2. Buying PrEP
3. Using PrEP
4. Getting PrEP without Medicare
5. Summary

This website is available in multiple languages

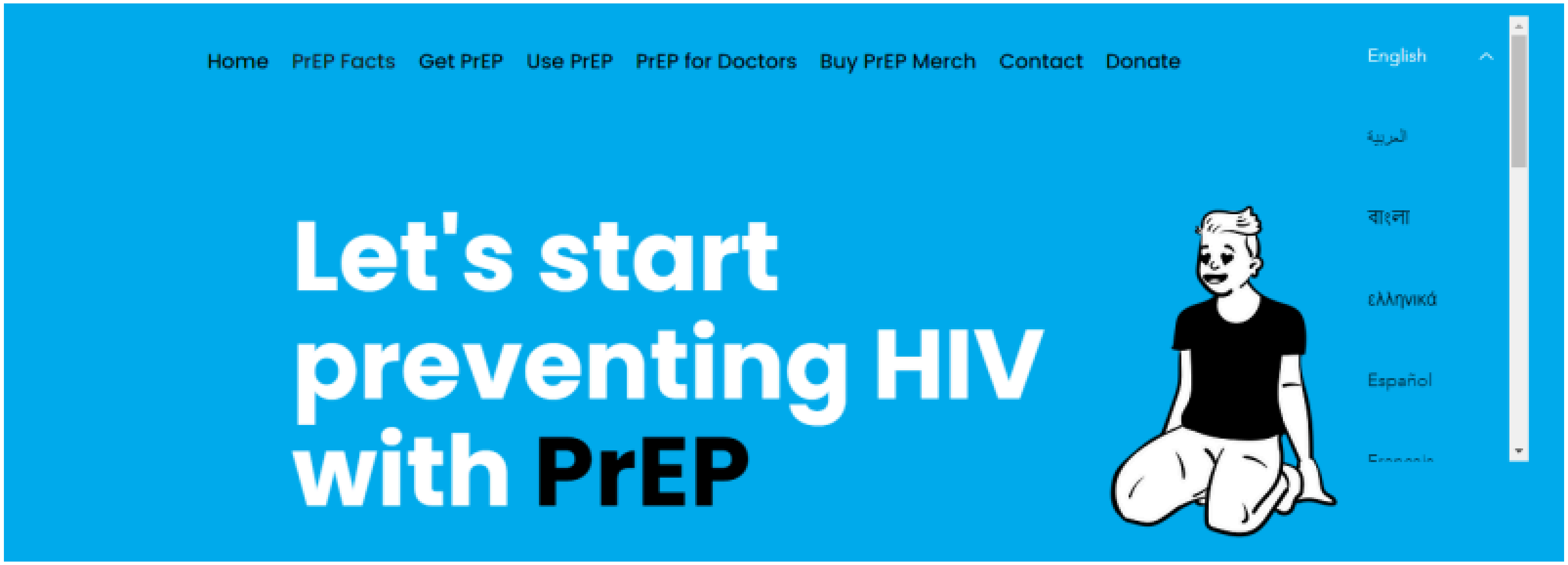

Please proceed to the next page, once you are ready to start the reading.

Please click all the links provided to read the articles

1. Starting PrEP
https://www.pan.org.au/start-prep
https://www.pan.org.au/find-a-doctor

After you finish reading, please come back to this survey to proceed to the next article.

2. Buying PrEP
https://www.pan.org.au/buy-prep-online
https://www.pan.org.au/free-prep

After you finish reading, please come back to this survey to proceed to the next article.

3. Using PrEP
https://www.pan.org.au/use-prep

After you finish reading, please come back to this survey to proceed to the next article.

4. Getting PrEP without Medicare
https://www.pan.org.au/no-medicare
After you finish reading, please come back to this survey to proceed to the next article.

5. Summary
https://www.pan.org.au/
After you finish reading, please come back to this survey to proceed to the next article.

Arm 2

The online media you have received is

an AUDIO DRAMA developed by the local community in Australia.

- There are 6 episodes of a 5 minute audio drama, which will take around 30 minutes to complete all episodes.
- There are accompanying scripts provided to assist individuals whose primary language is not English, facilitating a more comfortable listening experience.
- You are required to complete listening to all the episodes before answering the post-audio drama questions.
- Please be open to the content as it is well developed by the community.

Welcome to our audio drama, developed by and for the local community in Australia. Join us as we embark on a journey crafted with care and authenticity, featuring characters who are not actors, but individuals from our very own community.

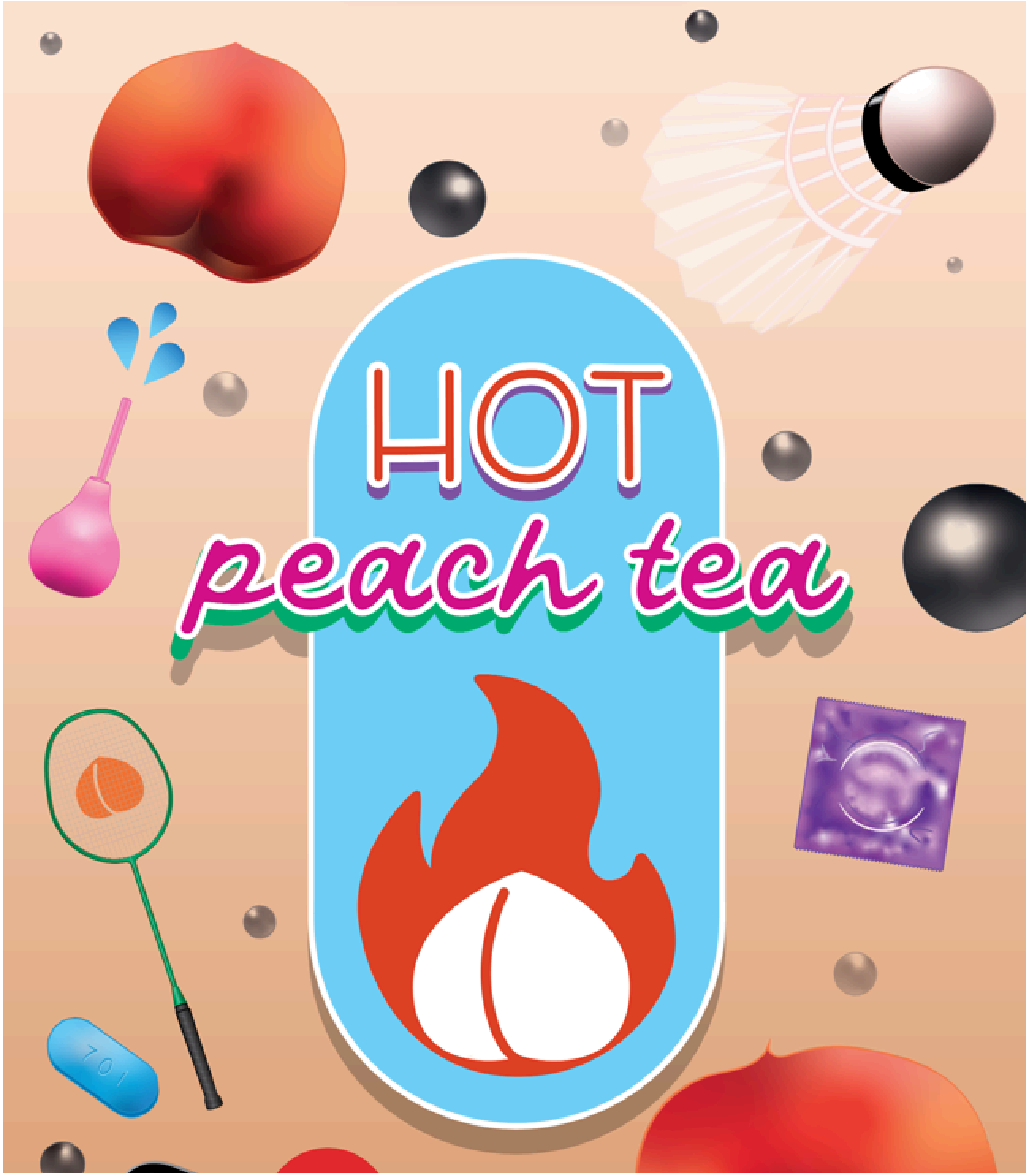
HOT
peach tea

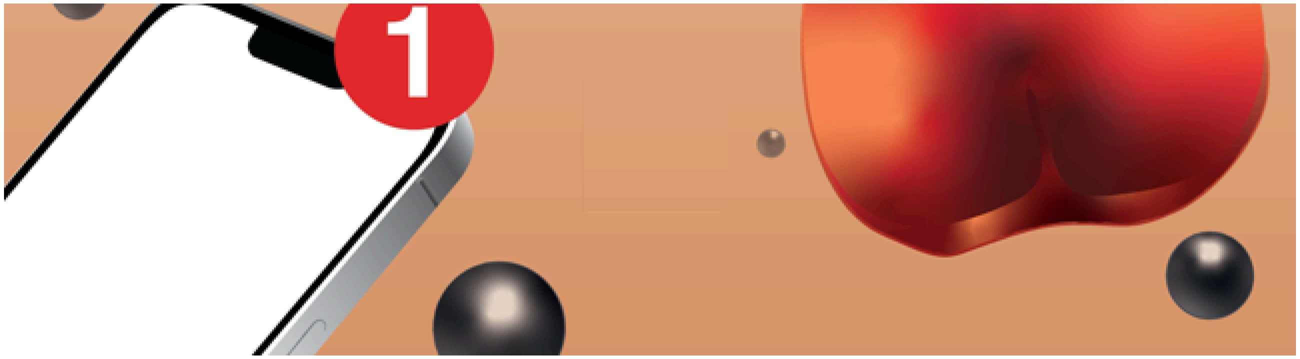

Synopsis

"Hot Peach Tea" follows the journey of Xiaolin, a 22-year-old Chinese newcomer to Australia, as he navigates the complexities of the gay scene in Melbourne alongside his friends Sunny and Derek. In the six compelling episodes, Xiaolin grapples with misconceptions and barriers surrounding PrEP (Pre-Exposure Prophylaxis) while exploring his identity and relationships within the queer Asian-born MSM community.

Episodes

1. He asked me if I was on PrEP... I said I douched
2. To PrEP or not to PrEP
3. How would you like to be PrEP
4. Sex after PrEP
5. No respect, No sex
6. In the end, community matters

Characters

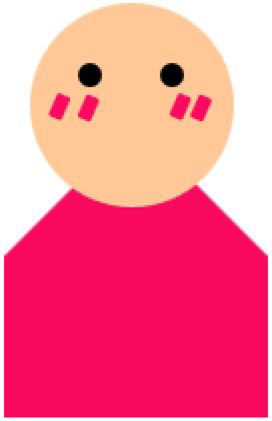

XIAOLIN
Gayby baby
22-year-old

A Chinese newcomer in Australia, explores his identity and sexual health with optimism, guided by friends, on a journey of self-discovery and empowerment.

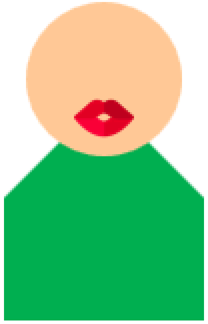

SUNNY
Queer sister
30-year-old

a Vietnamese non-binary cheeky sister who provides unwavering support and insightful guidance to Xiaolin throughout his journey.

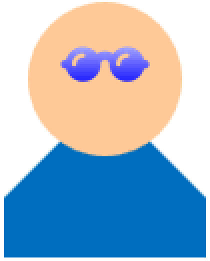

DEREK
Mentor
42-year-old

A Singaporean mentor, offers wisdom and reassurance to Xiaolin while navigating his own experiences.

Participants are required to complete listening to all six episode of the audio drama to be eligible for the $50 e-voucher. You can leave and come to back to this audio drama by opening the link with the same browser.

Episode 1: He asked me if I was on PrEP... I said I douched.

Kindly press the "PLAY" button to access the audio drama.

0:00 / 6:39

The accompanying script has been provided to ***assist*** *individuals whose primary language is not English, facilitating a more comfortable listening experience.*

Welcome to the audio drama HOT PEACH TEA – a production of Melbourne Sexual Health Centre.
This audio drama features sexual references for mature audiences.
You're listening to episode one:
"He asked me if I was on PrEP... I said I douched."

SCENE 1. BADMINTON COURT - AFTERNOON

XIAOLIN: Yes, yes. I *know* what you're thinking. Asians. Playing badminton. Of course. I don't have to be Australian or have my PR to know the stereotype. But it's been an amazing way to meet new people and we're quite good. Someone even calls us... something like, slay?? I need to look that up.

XIAOLIN: But I am talking too much and you don't even know me! Let me introduce myself - my name is Xiaolin, I'm 22 and I moved here six months ago from China after finishing my university studies in business management –

SUNNY: Xiaolin! You're forgetting some critical information here - your pronouns!

XIAOLIN: That's Sunny - I actually found out about the club through them. They

SUNNY: I don't really need an introduction but okay, I'll go. Actually, I'll just read my dating app profile: 29, Vietnamese, Pronouns: they/them, hot beverage flirt by day, and hot babing vers by night—

XIAOLIN: What's that even mean?

SUNNY: Honey, it means I make coffee for work and like both top and bottom when I play. Okay let me finish. I'm looking for: Right Now

XIAOLIN: Sunny!

SUNNY: What!? Okay okay, also looking for friends. Like you gayby baby, Xiaolin.

DEREK: Hey Sunny! Xiaolin! What are you doing!? You're both up!

SUNNY: Uhh love him but such a drag. Why's he so serious all the time?

XIAOLIN: And that's Derek. He's in his early 40s, Singaporean, married, and works in health. He's been here the longest, 15 years I think, and since I don't have any family here, he helps me out a lot. I go to Sunny for more gay chat and Derek for life chat because he's smart and nice... but yes, he can be too serious.

DEREK: Seriously! Are you here to play or what?

SUNNY: Oh my god! Did you tie your underwear when you tied the knot??

XIAOLIN: Alright, got to go. I'll catch you later, after the game. No wait - I'm busy. I'm getting picked up soon for a hook-up! Sunny helped me set up my profile and I've been chatting with this hot guy for a few days. His profile says Marco, 29 and open...? He must mean open-minded - that's nice. Anyway, it's my first time meeting up with a guy in Australia. I'm nervous but really excited…

SCENE 2. STREET - EVENING

XIAOLIN: Oh my god. Oh my god. Ahhh that was SO embarrassing. I can't believe... what just happened? I don't understand. Why am I such a newb?

XIAOLIN: Okay okay. Sunny and Derek will know, I'll ask them...

GROUP CHAT

XIAOLIN: Hey Sunny, Derek, are you still at the badminton court?? I just got out of the guy's car and walking back now. I'm freaking out.

SUNNY: Yeah honey, we're here!

DEREK: Xiaolin, you okay? What happened?

XIAOLIN: Uhh so you know, this guy Marco - he picked me up from badminton and we drove off somewhere. He stop somewhere quiet and dark and then we got right into it. We hardly talked before he started kissing me and taking my clothes off. Before I knew it, we were naked. It was sexy, I guess, but it was happening so quickly... then suddenly he stopped and was laughing at me. Then he kicked me out of the car! I'm so embarrassed and confused. What did I do

wrong?

DEREK: Really? That's crazy! Did he say anything before he kicked you out??

XIAOLIN: Oh there was something... he asked me if I prepped and I said I douched... Did I say the wrong thing?

SUNNY: Oh honey - you didn't do anything wrong. I think you misunderstood him. He was asking you if you took PrEP not if you prepped.

XIAOLIN: Uhh... what's that??

DEREK: PrEP is pre-exposure prophylaxis. They're pills you can take to protect yourself from getting HIV.

XIAOLIN: What?? I didn't even know that exists. Do you take it Sunny, Derek?

SUNNY: Yeah I do, boo! I've been taking it for years – it means I can have all the sex I want without getting HIV. I take it daily but I have friends who take it on-demand or periodically.

DEREK: Uhh I don't want to overload you right now... Xiaolin, let's meet up now when you get back
to the badminton court. We can go get boba and tell you more about PrEP. Now, the main thing is you didn't do anything wrong. Who kicks someone out like that? You may have douched but that guy Marco, if that's even his real name, sounds like the real douche here.

SUNNY: Oooh, I like it when Derek gets spicy! Yeah I mean, was he even hot? I want all the juicy tea.

XIAOLIN: Well, yeah he's hot! I saw when he was driving he has this strange tattoo on his wrist... I think I noticed it because I've been thinking I want one. Anyway, I'll tell you more soon when I see you!

Upcoming episode:

XIAOLIN: My parents in China have access to my bank account — what if they see a transaction for PrEP and find out?? I'd literally be dead.

HOT PEACH TEA is supported by funding from the Australian National Health and Medical Research Council and is produced by staff from the Melbourne Sexual Health Centre.
This audio drama is for educational information purposes only and is not a substitute for professional health advice. To learn more about the Melbourne Sexual Health Centre, please visit our website: www.mshc.org.au.

Upcoming episode:

XIAOLIN: My parents in China have access to my bank account — what if they see a transaction for PrEP and find out?? I'd literally be dead.

HOT PEACH TEA is supported by funding from the Australian National Health and Medical Research Council and is produced by staff from the Melbourne Sexual Health Centre.
This audio drama is for educational information purposes only and is not a substitute for professional health advice. To learn more about the Melbourne Sexual Health Centre, please visit our website: www.mshc.org.au.

Episode 2: TO PrEP OR NOT TO PrEP?

Kindly press the "PLAY" button to access the audio drama.

0:00 / 4:46

*The accompanying script has been provided to **assist** individuals whose primary language is not English, facilitating a*

more comfortable listening experience.

SCENE 1. XIAOLIN'S BEDROOM - NIGHT

XIAOLIN: Health anxiety dot com… Better Health Advice dot net... Ending HIV dot org?? Australian PrEP guidelines... CraftyCry on Reddit says side effects are more than they say... but OpenTwink says there's hardly any? And it says here you can take PrEP daily but also on demand... What does that mean?

XIAOLIN: Uhh!! I don't know what to believe! I need to sleep on it.

SCENE 2. BADMINTON COURT - DAY
SUNNY: You're playing like my ông ngoại!

XIAOLIN: Your grandpa doesn't have moves like this...

DEREK: Righto! Xiaolin's game. Sunny, boba's on you today.

SUNNY: …aaand there's grandpa.

XIAOLIN and **SUNNY:** hahaha.

SCENE 3. BADMINTON COURTSIDE - MOMENTS LATER

SUNNY: Alright boo, spill – it's been a week since your hook-up with Marco. How you feeling?

DEREK: And any thoughts on trying PrEP?

XIAOLIN: Ahh I'm still feeling embarrassed... now so confused about it all.

SUNNY: Aww what's making you confused, honey?

XIAOLIN: I did all this research last night, read all these health sites and forums with people who take PrEP, and there's so much different advice... I don't know enough but I don't think it's for me.

DEREK: That's understandable Xiaolin, though I think — oh! Is that the time? We only have this court for another 10 minutes!

SUNNY: Ooo okay how about a rally? You **serve** us this round gayby baby, and you can also swing your concerns our way!

XIAOLIN: Oh, yep!

SCENE 4. BADMINTON COURT - AFTERNOON

XIAOLIN: Ready!?

SUNNY: Yas Queen!

DEREK: Serve already!

XIAOLIN: Okay, I don't have Medicare… and it's expensive to see
the doctor and get testing, and then the cost of PrEP - I'm no Crazy Rich Asian!

DEREK: Xiaolin, consultations and tests are actually free at some clinics and you can order the medication online at reasonable prices — it ranges from $20-$30 per month.

XIAOLIN: Oh okay...!? Well, anyway I feel PrEP is for the more promiscuous of gays.

SUNNY: This one's mine, Derek! Babe. That's a myth based in slut-shaming, yeah? Using PrEP doesn't increase frisky behaviour and no way it's only for those who are more sexually active. It's empowerment. It's about the right to health and to tools available for protecting ourselves from HIV. Making sense, sweetie?

XIAOLIN: Ahuh...

SUNNY: Stunninnnng!

XIAOLIN: Hmm what about my ginseng?? I read that PrEP mixes badly with Chinese herbal medicine??

DEREK: Oh PrEP rarely interacts with or has an effect on common medication or even Chinese herbs. I've got a friend

who takes both – they see a Chinese herbalist and also a GP about it. I suggest discussing it with your doctor and a pharmacist as well, to be sure. It's more work, but worth it!

XIAOLIN: Okay... great! One last thing though. My parents in China have access to my bank account — what if they see a transaction for PrEP and find out?? I'd literally be dead.

DEREK: They'll never know —

SUNNY: — we can help you set up an account *RIGHT NOW* in Australia so they can't see!

DEREK: Wait. Hang on, I think you're getting ahead of yourself, Xiaolin. At the end of the day, your health is the most important thing and you can cross any bridge if and when you get to it! Making a decision based on other people's experiences isn't going to help you. I really think getting a sexual health check is best - it's important to get professional input specific to your own circumstances. I'll even come with you on my day off for moral support! What do you say?

XIAOLIN: Sounds great. Thank you — both of you.

SUNNY: Yesss queeeeen! You show that health check what Xiaolin's made of!

Upcoming episode:

XIAOLIN: What if I have HIV? And the government gets my results and I get deported, then my parents will find out!!

HOT PEACH TEA is supported by funding from the Australian National Health and Medical Research Council and is produced by staff from the Melbourne Sexual Health Centre.

This audio drama is for educational information purposes only and is not a substitute for professional health advice. To learn more about the Melbourne Sexual Health Centre, please visit our website: www.mshc.org.au.

Episode 3: HOW WOULD YOU LIKE TO PrEP?

Kindly press the "PLAY" button to access the audio drama.

0:00 / 6:55

*The accompanying script has been provided to **assist** individuals whose primary language is not English, facilitating a more comfortable listening experience.*

--

SCENE 1. SEXUAL HEALTH CLINIC - MORNING

XIAOLIN: *'I'm so late to meet Derek at the Sexy Health Clinic... he suggested a health check and get some professional advice about taking PrEP but I'm feeling not so slay, as Sunny would say. What if I already have HIV? Or another STI??'*

DEREK: Xiaolin? Is that you under the hat and mask?

XIAOLIN: Oh you can tell? I thought this was a master disguise, to hide my identity...

DEREK: Oh there's nothing to be embarrassed about! Getting a health check is completely normal and 100% confidential, I promise.

XIAOLIN: Okay I'll take it off. But I'm still nervous.

DEREK: That's okay - I'll be with you every step of the way, Xiaolin.

XIAOLIN: Thank you, Derek.

SCENE 2. SEXUAL HEALTH CLINIC WAITING ROOM - MOMENTS LATER

XIAOLIN: *'This room is full of people looking at me —- wait. No one is looking at me.I'm staring. Okay, deep breaths Xiaolin. What if someone recognises me? It was so nice of Derek to come support... What if someone recognizes him? Okay calm down. Look around the room just to make sure there's no one here who know me.'*

XIAOLIN: *'Bald person with glasses - no. Older person with big smile - no. Hmmm, not them, not them, not them—-'*

DOCTOR: XIAOLIN, September?

DEREK: That's you! You'll be fine. I'll be right here when you're done and we can debrief if you'd like.

XIAOLIN: Okay, here I go...

SCENE 3. EXAMINATION ROOM - MOMENTS LATER

DOCTOR: Lovely to meet you, Xiaolin - I'm Milly, your doctor for today. I understand you're here for a sexual health check and PrEP?

XIAOLIN: Ummm yes.

DOCTOR: You seem a little nervous which is completely understandable but you have nothing to worry about. I'll start by asking you some preliminary questions - can you tell me who do you normally have sex with? Males, females, or both?

XIAOLIN: I am gay...?

DOCTOR: And how do you usually have sex?

XIAOLIN: Ummm... up my bum? And up other people's bums.

DOCTOR: So anally - great. How many sexual partners have you had in the past three months?

XIAOLIN: Only the guy I tried to hook up with a couple weeks ago but we didn't have sex - it's why I think I want PrEP. But I'm no playboy!

DOCTOR: Xiaolin, there's no shame in taking PrEP or having multiple sexual partners, as long as it's consensual for everyone and works for you. Last question, have you had any symptoms like discharge, burning when you pee, itchiness?

XIAOLIN: Ummm... no.

***XIAOLIN:** 'Is that right? I would remember if there was anything like she is saying... but my memory can be terrible sometimes and lately so much going on with–-'*

DOCTOR: Xiaolin? Are you still with me?

XIAOLIN: --yes, sorry Doctor.

DOCTOR: Great - I just want to say again that you're doing the right thing by getting tested. Patients sometimes regret delaying the test and really, there's nothing to lose. The sooner you test, the sooner you can be in a better position to understand your health and if needed, take treatment. These days, HIV is very manageable.

XIAOLIN: Thank you Doctor... Can you tell me about PrEP? All the websites confuse me but after talking to friends, I think I want it.

DOCTOR: Of course - PrEP stands for pre-exposure prophylaxis. It involves people who don't have HIV taking medication to protect themselves and prevent HIV infection. When used as prescribed, PrEP is up to 99% effective at preventing HIV infection, particularly for men who have sex with men.

XIAOLIN: Ahh...

DOCTOR: But to be able to take it we need to know you do not have HIV first. So, I'm going to send you to get some tests in the next room with one of our nurses - we'll be doing a blood test to check for HIV and syphilis, and do a urine test, a throat and bum swab for other common STIs like chlamydia and gonorrhoea. Do you have any questions?

XIAOLIN: Uh... Can my parents back in China find out my results? They don't even know I'm gay.

DOCTOR: Not at all, Xiaolin. Unless you tell them. Your results are kept private and confidential.

XIAOLIN: Oh great. That's good to know, thank you Doctor Milly!

SCENE 4. SEXUAL HEALTH CLINIC WAITING ROOM - MOMENTS LATER

DEREK: How'd you go?

XIAOLIN: Derek! It wasn't as scary as I thought! I am worried about the results and what if it affects my visa or my parents find out. But the doctor said it's confidential.

DEREK: That's right - it's better you find out sooner than later and if necessary, you can get treatment. It shows that you are managing it and will be better for you visa-wise and of course, your health.

DEREK: Okay - why don't you go pee in the container they gave you and do your swabs while you wait for the nurse to take your blood? The toilet is down the hallway, just around the corner. I'll wait for you back here.

XIAOLIN: Okay!

XIAOLIN: *'I'm feeling so much better about everything now. So glad Derek is here to guide me.'*

XIAOLIN: Ohh this line is long.

WOMAN: Ughh lines hey? I can't stand them.

XIAOLIN: *'Hey this lady has a tattoo on her wrist... that looks just like the same one as Marco, douche man. Why do they have the SAME tattoo?? Are they together? Sunny says people on the apps aren't always true. Are they in a relationship and he's cheating on her? And what if I have HIV and I gave it to Marco who could give it to her!'*

XIAOLIN: *'Am I staring? Don't stare. I'm staring! XIAOLIN, STOP STARING!'*

WOMAN: What are you looking at?? Oh, my tattoo. For a moment, I thought my fly was undone.

XIAOLIN: Uh yeah... I like it.

WOMAN: Thanks! I got it a few years ago. It was a silly decision I made with my *then* boyfriend, James, to get matching tattoos while we were traveling through Italy.

XIAOLIN: ‘OH MY GOSH that’s such a relief - that means I’m no homewrecker... also she said James, not Marco. But Sunny did say these guys can use fake names...’

WOMAN: We were young then... it was stupid. But at least, we’re married now so it’s for life!

XIAOLIN: **WHAT.**

Upcoming episode:

XIAOLIN: Okay remember last month I saw that woman at the clinic with the same tattoo as douche man Marco? And she said she has a matching tattoo with her husband James... well, this morning I was scrolling away and guess who I saw?! Same photos as Marco but this time he’s Luka.

HOT PEACH TEA is supported by funding from the Australian National Health and Medical Research Council and is produced by staff from the Melbourne Sexual Health Centre.

This audio drama is for educational information purposes only and is not a substitute for professional health advice. To learn more about the Melbourne Sexual Health Centre, please visit our website: www.mshc.org.au.

Episode 4: “SEX AFTER PrEP”

Kindly press the "PLAY" button to access the audio drama.

0:00 / 6:04

*The accompanying script has been provided to **assist** individuals whose primary language is not English, facilitating a more comfortable listening experience.*

SCENE 1. INT - XIAOLIN'S BEDROOM - MORNING

XIAOLIN: (*Confidently*) Oh heyyyy! Yes, you, listener! It's been a whollleee lunar cycle since we last chatted. I got the results of my sexual health check and guess what?! This boo is all clear! HIV negative and no STIs so I got prescribed PrEP! At first I had a little bit of nausea and tiredness but they stopped after a few days and it's fine since. I was a little scared but Sunny spoke to me saying the minor side effects from PrEP are an easy trade off for protection against HIV and pieces of mind. They so wise like my grandma, Po Po.

XIAOLIN: So every week, I've been trying to go on a date...

GAY MAN #1: Oh you're on PrEP too? Wanna come back to mine for some fun?
GAY MAN #2: Thanks for the fun night! Do you want to cum inside?
GAY MAN #3: Konnichiwa! Your skin is sooo smooth and delicious. I so badly want your sushi roll...

XIAOLIN: Okay, not all of my dates went well. I'm not even Japanese! But Sunny's shown me to block racist guys on the app. ANYWAY my point is I feel so empowered now! I use PrEP when I play, to keep HIV at bay and condoms too so STIs stay away! I came up with that - Sunny thinks it's cheese so I can tell they like it. Anyway – all the hookups are nothing like that first time with Marco...

XIAOLIN: But I have been thinking lately... Am I becoming the person I didn't want to be? Has PrEP made me a slut..?

XIAOLIN: No, no, PrEP has given me more options to explore my sexuality and be safe - and there's nothing wrong with that! Just like Sunny and Derek said. I wonder who is around right now, let's check the grid...

XIAOLIN: Hmm not today... not you... you pretty but... too pretty. Oh you're cute... yes to you... oh you would be a good match with *that* guy... OH! What!! It's Marco!! But it says Luka? Hmmm... Marco? Luka? They both sound Italian? Clinic tattoo woman did say they got matching tattoos in Italy. Is it just coincidence? I NEED to know. And maybe we can re-do last time with a happier result...

SCENE 2. SUBURBAN STREET - LATER, AFTERNOON
XIAOLIN: Oh my god. Oh my god. Ahhh that was SO juicy. I NEED to tell Sunny and Derek.

<u>GROUP CHAT</u>
XIAOLIN: YOU TWO. This gaysian has some tea! I'll catch you up when we get boba after badminton tonight!

DEREK: Sure thing, Xiaolin.

SUNNY: YASSSSS. Call me an Asian Karen at yum cha asking for the manager because I CANNOT WAIT.

SCENE 3. BOBA TEA SHOP - LATER, EVENING

DEREK: annnnd the milky boba for Xiaolin...Great game gang. Sunny, I think you could work on your swing—

SUNNY: -Oh shut up old man! Xiaolin, we have our tea cups ready. SPILL.

XIAOLIN: Okay remember last month I saw that woman at the clinic with the same tattoo as douche man Marco? And she said she has a matching tattoo with her Husband James... well, this morning I was scrolling away and guess who I saw?! Same photos as Marco but this time he's Luka!

DEREK: ... I'm not following...

XIAOLIN: Marco, James and Luka are all the same person!

SUNNY: Wait. You went to see him?!

XIAOLIN: Yes - detective Xiaolin was on the case. Also I didn't get to taste test last time...

SUNNY: That's my gayby!

XIAOLIN: I was direct and asked if his real name is James. He was awkward at first but yes!

DEREK: What?! He's using fake names to cheat on his wife?

XIAOLIN: Well, he said him and his wife are open... I wasn't sure what that mean and he explain they are happily in a committed relationship but like to have fun with other people too. So I'm no homewrecker!

SUNNY: Oh that's so great hun!

XIAOLIN: And before we kissy kissy I told him that even though I'm on PrEP I want to keep safe from other STIs with

condoms and he agreed. Such great chatting and Feeling safe together. It was amazing!

XIAOLIN GROANING IN DISCOMFORT

SUNNY: What's wrong? You okay, Xiaolin?

XIAOLIN: Oh —- I don't think I should have got milky boba. I need to go to toilet! Be right back.

XIAOLIN QUICKLY STANDING UP AND RUSHING AWAY

DEREK: Wow! What a rollercoaster. I'm so happy for Xiaolin though. It seems like he's finding himself – his confidence is glowing like the full moon! I'm proud of him.

SUNNY: A Leo full moon baby! He's in his damn slay era. It's amazing how sexually empowered he seems and that he's doing it safely. We. LOVE. A safe twink. I think we should be proud of ourselves for helping. I mean it's all Xiaolin but you know... little pats on the back...We make great parents, Derek.

DEREK: Uhhh Sunny I don't think —-

XIAOLIN RUSHING BACK TO TABLE

XIAOLIN: (*Nervously*) Ummm... I'm confused.

SUNNY: What's wrong, boo?

XIAOLIN: I don't understand... there's cum in my underwear..

Upcoming episode:

SUNNY: Babe, I think you've been stealthed. You and James agreed to use a condom but I think he lied about putting it on or removed it without your consent.

HOT PEACH TEA is supported by funding from the Australian National Health and Medical Research Council and is produced by staff from the Melbourne Sexual Health Centre.
This audio drama is for educational information purposes only and is not a substitute for professional health advice. To learn more about the Melbourne Sexual Health Centre, please visit our website: www.mshc.org.au.

Episode 5: NO RESPECT, NO SEX

Kindly press the "PLAY" button to access the audio drama.

0:00 / 6:30

*The accompanying script has been provided to **assist** individuals whose primary language is not English, facilitating a more comfortable listening experience.*

This episode contains discussion of sexual violence and stealthing. Listener discretion is advised. For resources and support:

- 24 hour free counselling in Australia, call 1800RESPECT or Lifeline on 13 11 14.
- Melbourne Sexual Health Centre https://www.mshc.org.au/clinics-services/our-services/counselling
- QLife https://qlife.org.au/get-help/

SCENE 1. BOBA TEA SHOP - EVENING

XIAOLIN: Ummm... I'm confused.

SUNNY: What's wrong, boo?

XIAOLIN: I don't understand... there's cum in my underwear...

SUNNY: What? But honey, I thought you used a condom?

XIAOLIN: We did! But it's definitely cum - I actually felt it dripping before but I thought it was sweat.

SUNNY: NO WAY. This is just...

XIAOLIN: What?!

SUNNY: Babe, I think you've been stealthed. You and James agreed to use a condom but I think he lied about putting it on or removed it without your consent. It's a form of sexual violence... and a crime.

XIAOLIN: Ohhhh...

SUNNY: I'm sorry this happened to you, boo.

DEREK: Xiaolin..?

XIAOLIN: Uh yeah. So... what should I do?

SUNNY: Well you could take legal action if you wanted to. It's entirely up to you.

XIAOLIN: I don't know... I feel embarrassed all over again.

DEREK: If you *were* stealthed, there's absolutely no shame in being a survivor of sexual violence.

SUNNY: No shame at all, hunny. You've done nothing wrong. Derek and I will always be here to support you!

XIAOLIN: Thank you.

SUNNY: Disrespect of consent and boundaries – unfortunately it happens. I've had to deal with one *too* many toxic tops that assume I'm submissive just because I'm Asian. Such bullshit! Not only because of that gross masc energy but

mostly because I'm a proud Viet-switch!

DEREK: That's awful Sunny - I'm sorry.

SUNNY: You know what? While I'm at it, *SCREW* those dating app profiles that say (*emphatically*) *no to everything delicious and beautiful like fats, femmes, and Asians*. More like "No respect, *NO SEX*!"

DEREK: Absolutely!

SUNNY: Anyway, thanks for listening to my TED talk. I know this isn't about me but this stuff gets me mad.

DEREK: No, Sunny - it's about all of us. Thank you for sharing.

SUNNY: I'm gonna get more boba - you sweeties want anything?

DEREK: Nah, thanks.

XIAOLIN: No thank you, Sunny.

SUNNY GETS UP AND WALKS AWAY

DEREK: Xiaolin, I don't want to downplay the significance of what happened but to offer another point of view... condoms break sometimes. Over the years, through personal experience as well as my work in health, I know accidents do happen. That doesn't mean it doesn't suck to deal with but it's possible James didn't do this on purpose. At least you and James are both on PrEP. You haven't missed any doses, have you?

XIAOLIN: No, I haven't. That's good, right?

DEREK: That's great. Hey, I've got an idea - why don't you check if he's still online?

XIAOLIN: Why?

DEREK: It might seem scary but you could message him asking to chat? I really think you need to find out more before jumping to any conclusions. Particularly if he

hasn't blocked you.

XIAOLIN: Hmm okay. Let me see...

XIAOLIN: He's not blocked me! Okay here we go... Hey James... I want to talk to you - call me on 0412 7 XXXX Okay - done... Hopefully he calls soon.

SCENE 2. XIAOLIN'S BEDROOM - MORNING

XIAOLIN: 'Oh my GOD it's been 17 hours and feels like I've been lying in bed forever... why hasn't James called yet?? I don't know what to do... should I go to his house?? *NO* that's a bad idea... I know Sunny and Derek said I did nothing wrong but I feel... so much shame... what if my parents find out?? Is this what I get for being more sexually active?

PHONE BUZZING

XIAOLIN: IT'S JAMES.

PHONE CALL
JAMES: Xiaolin?

XIAOLIN: ...heyy.

JAMES: Hey. I know what you're wanting to talk about - while I was cleaning up after yesterday, I realised that the condom broke.

XIAOLIN: Oh... so it did break?

JAMES: It was a complete accident but I know how it seems given I left you in the dark for so long. I was scared of what you might think of me, especially after the first time we tried to hook up. I'm *so* sorry for not calling you sooner.

XIAOLIN: Oh okay...

JAMES: I handled this all poorly... Again, I'm really sorry, Xiaolin.

XIAOLIN: I understand, it happens. It's good we chatted before about PrEP so at least we safe from HIV.

JAMES: Absolutely. Saying that, I think we should both get a sexual health check just to make sure.

XIAOLIN: Good idea. Thank you for being honest, James.

JAMES: Thank *you*. Take care now, let me know how you go.

END OF THE CALL

XIAOLIN: Ughhhggghhhh. Thank GAWD! I jumped conclusions with James, just like Derek is always warning me about. It was great we could talk like that... but not everyone is like that. I think this has inspired me to be more upfront, honest and say exactly what I want and how I'm feeling. Yeah... I want to have courage to have these conversations rather than feel fear of being sexually or socially rejected. Hmm. I should change my app profile bio. No... respect... no... sex.

Upcoming episode:

DEREK: I avoided sharing my PrEP status when you first came to us Xiaolin because even though he's proudly living with HIV, there's still a strong stigma...

HOT PEACH TEA is supported by funding from the Australian National Health and Medical Research Council and is produced by staff from the Melbourne Sexual Health Centre.

This audio drama is for educational information purposes only and is not a substitute for professional health advice. To learn more about the Melbourne Sexual Health Centre, please visit our website: www.mshc.org.au.

Episode 6: IN THE END, COMMUNITY MATTERS

0:00 / 7:48

Kindly press the "PLAY" button to access the audio drama.

The accompanying script has been provided to ***assist*** *individuals whose primary language is not English, facilitating a more comfortable listening experience.*

SCENE 1. BOBA TEA SHOP - AFTERNOON

XIAOLIN: I can't believe it's been over six months since we started the world's best group chat: Hot Peach Tea.

SUNNY: It's giving friendship, it's giving love, it's giving Gaysian, sweet gayby baby, sexual empowerment, slay...

DEREK: It's giving... Wait, this is dumb you two, we're sitting right next to each other.

XIAOLIN AND SUNNY CHUCKLE.

SUNNY: Okayy *UNCLE*.

XIAOLIN AND SUNNY CHUCKLE AGAIN.

XIAOLIN: Me from six months ago wouldn't believe how good I feel now! Taking control of my sexual health through PrEP makes me feel so empowered. And this confidence is flowing into all parts of life... even work!

SUNNY: Babe! *EVEN* badminton.

XIAOLIN, SUNNY and DEREK CHUCKLE.

DEREK: Absolutely! Your swing is on fire.

XIAOLIN: But taking pill every day and getting sexual health check every three months are annoying... I've been thinking about stopping taking PrEP... or it would be easier if I could just get PrEP from a pharmacy and never have to go to the clinic for testing.

DEREK: As always, it's entirely your choice...

SUNNY: And I understand the pill fatigue...

DEREK: But there are different ways to use PrEP like on-demand where you take it only when you're at risk of getting HIV or periodically...

SUNNY: And of course it's always important to consider the safety of your partners and yourself if you're going to keep dating, ya know? That's why it's important to get ongoing HIV and STI checks every three months.

XIAOLIN: Yeah...

SUNNY: *This* qweeen takes PrEP to make sure, no matter what, I'm safe and my community is safe. The greater good! It's such peace of mind for when I want a hook up, planned or surprise romantic—-

DEREK: Actually, I don't take PrEP because U=U.

XIAOLIN: What's U=U?

DEREK: It means undetectable equals untransmittable. My husband is HIV positive.

XIAOLIN: Oh? Really?!

DEREK: I avoided sharing my PrEP status when you first came to us Xiaolin because even though he's proudly living with HIV, there's still a strong stigma...that and I didn't have a chance to bring it up sooner with all your *drama* lately...

DEREK CHUCKLES TO HIMSELF. XIAOLIN AND SUNNY JOIN.

SUNNY: Tell him more now, I think he's ready for it.

XIAOLIN: Yes, drama is off the table... *for now.*

DEREK: So my husband Carlos has undetectable levels of HIV which means it is untransmittable. He can't transmit HIV to me. You might have seen the U=U campaign about it? And we're monogamous so there's no risk to anyone else. I don't take PrEP and am safe but that's a decision I made with Carlos when we committed to each other 15 years ago and only after consulting my doctor. This is all to remind you that there are many different ways to go about safe sex and to do what is right for you and your circumstances. Ultimately, choices should be informed and supported by advice from health professionals.

SUNNY: Preach it, sister!

DEREK: Not sure if this has helped, Xiaolin?

XIAOLIN: It has... thank you for sharing Derek. And thank you Sunny. You're both amazing. Since I want to keep dating different, *sexy* people...

DEREK AND SUNNY CHUCKLE.

XIAOLIN: Taking one pill a day for pieces of mind really isn't that bad. It's kind of like badminton training. Using PrEP isn't just protection for me but also my sexual partners - and it makes me keep healthy and a better team player!

SUNNY: That's *So* Raven. We. *LOVE.* A sports queen who slays at sexual health.

XIAOLIN: I'm going to get a new script now. See you next week!

SCENE 2. SEXUAL HEALTH CLINIC - MORNING

XIAOLIN: *'I'm so grateful to have Sunny and Derek. They so sweet and our friendship has helped me grow from Gayby Baby into proud Gayby Twink. It would have been so hard without their support... I wonder if I could be that for someone one day? What I think would slay is a queer badminton club that welcomes people new to Australia... with room for oldies like Derek too! Hmm... that would be fun.'*

XIAOLIN: *'Okay back again - it feels like so long since my first time coming to the clinic. And there's a person here that*

looks like me but much younger... a Baby Gayby Baby! They have mask and are pacing up and down the footpath... they look nervous. I hope they're okay. Reminds me of when I first came here - lucky ducky I had Derek. Hmm... I'll go over and check in with them.'

XIAOLIN: Hey.

SI-EN: Hello.

XIAOLIN: I'm Xiaolin. I love your buzz cut.

SI-EN: Oh! Thank you. I shaved when I got to Australia. (*Pause*) It feels more like me.

XIAOLIN: That's great! So this is your first time to the clinic?

SI-EN: Yes... you can tell?

XIAOLIN: I was nervous the first time. But I had a friend remind me I was doing the right thing and getting a sexual health check is completely normal. It's even empowering!

SI-EN: Oh... okay. Thank you... *Xiaolin*..? Do you have an English name you use too? My name is Si-En but I worry people can't say it. So I use Ben.

XIAOLIN: I get that. I felt like that when I arrived too but I love my Chinese name - it means little forest! How could I not. It's entirely your choice though, whatever you feel comfortable with. I have these wonderful friends that encourage me all the time there's no one size fits everything - you do what's right for you.

SI-EN: They sound nice!

XIAOLIN: Oh! Do you like badminton?

SI-EN: ... you asking because I'm Asian?

XIAOLIN: No! Well, maybe! I'm in a club full of beautiful, queer, inclusive Asians - we practice on the courts a few streets over! It would be great to see you there!

SI-EN: What's the name of the club?

XIAOLIN: We're called HOT PEACH TEA!

HOT PEACH TEA is supported by funding from the Australian National Health and Medical Research Council and is produced by staff from the Melbourne Sexual Health Centre.
This audio drama is for educational information purposes only and is not a substitute for professional health advice.
To learn more about the Melbourne Sexual Health Centre, please visit our website: www.mshc.org.au.

This Audio drama is...

Produced by:
Melbourne Sexual Health Centre

Written by:
Rae Choi
Joshua Tate

Directed by:
Alexander Luciano Hudson
Riley Siu
Tiko Istiko
Shushen Feng

Music by:

Rudy Bilani

Audio Editor:

Riley Siu

Voice Cast:

Xiaolin: Shushen Feng

Sunny: James Tapa

Derek: James Seow

Additional Voices: Phyu Mon Latt, Nyi Nyi Soe, Alicia King, Rudy Bilani, Alexander Luciano Hudson, Riley Siu, Tiko Istiko, Jason Ong

Special Thanks:

Australian National Health and Medical Research Council

Jason Wu

Post-survey

Your almost done!

After the online media, we would like to know the changes in how likely you would be to use PrEP and knowledge about PrEP.

Please indicate the extent to which the online media has influenced your thinking about PrEP

	Strongly disagree	Somewhat disagree	Neither agree nor disagree	Somewhat agree	Strongly agree
The online media has increased my awareness of PrEP as an effective HIV prevention method.	○	○	○	○	○
I am more likely to consider using PrEP for HIV prevention based on what I've learned from the online media	○	○	○	○	○
The online media has positively influenced my attitude towards PrEP for HIV	○	○	○	○	○

How likely are you to do the following during the next 3 months?

	Definitely Will Not Do	Probably Will Not Do	Probably will do	Definitely will do
I will talk to a health care provider about PrEP	○	○	○	○
I will seek out more information about PrEP.	○	○	○	○

	Definitely Will Not Do	Probably Will Not Do	Probably will do	Definitely will do
I will get a prescription for PrEP.	○	○	○	○

For each of the following questions, please choose True (T), False (F), or Don't Know (DK). If you do not know, please do not guess; instead, please choose Don't Know.

	True	False	Don't know
PrEP is a pill you can take after sex to reduce your risk of becoming infected with HIV.	○	○	○
There may be some herbal medicine that can reduce the effectiveness of PrEP in preventing HIV.	○	○	○
PrEP can be used to prevent sexually transmitted infections like gonorrhea, chlamydia, syphilis, and herpes.	○	○	○
PrEP can be taken by people who already have HIV.	○	○	○
If you start taking PrEP, you will have to take it for the rest of your life.	○	○	○
You need insurance or Medicare to access PrEP in Australia.	○	○	○
In Australia, I can get PrEP at a pharmacy without seeing a doctor.	○	○	○
You must take an HIV test every 3 months while taking PrEP in Australia.	○	○	○

	True	False	Don't know
There are many serious side effects of taking PrEP.	○	○	○
Only daily PrEP can lower the risk of getting HIV from sex.	○	○	○

Do you agree or disagree with the following statement?

	Strongly disagree	Somewhat disagree	Neither agree nor disagree	Somewhat agree	Strongly agree
PrEP is effective at preventing HIV.	○	○	○	○	○
People who take PrEP are responsible.	○	○	○	○	○
Taking PrEP is safe.	○	○	○	○	○
It would be no trouble to take PrEP every day.	○	○	○	○	○
The government makes certain that drugs like PrEP are safe.	○	○	○	○	○
People without Medicare cannot access PrEP in Australia.	○	○	○	○	○
PrEP is not affordable for people without Medicare.	○	○	○	○	○
PrEP cannot be taken with any herbal medicine.	○	○	○	○	○

	Strongly disagree	Somewhat disagree	Neither agree nor disagree	Somewhat agree	Strongly agree
PrEP is for only highly sexually active people.	○	○	○	○	○
Being on PrEP is stigmatising.	○	○	○	○	○

Audio drama

Would you recommend this audio drama to others?

- ○ Yes
- ○ No

What are your general impression of the audio drama?

	Strongly disagree	Somewhat disagree	Neither agree nor disagree	Somewhat agree	Strongly agree
I feel the content shown in the audio drama was relevant to me.	○	○	○	○	○
I learned information in the audio drama that will help me in preventing HIV.	○	○	○	○	○

	Strongly disagree	Somewhat disagree	Neither agree nor disagree	Somewhat agree	Strongly agree
I did not feel the audio drama gave me any information I did not already know.	◯	◯	◯	◯	◯
I felt the content of the audio drama was culturally appropriate.	◯	◯	◯	◯	◯
I think the content of the audio drama can help newly-arrived Asian born men better prevent HIV.	◯	◯	◯	◯	◯
I think this audio drama is the best way to encourage newly-arrived Asian born men who have sex with men to learn about and start taking PrEP.	◯	◯	◯	◯	◯

Your feedback on the audio drama will help us improve its quality and content.

	Extremely dissatisfied	Somewhat dissatisfied	Neither satisfied nor dissatisfied	Somewhat satisfied	Extremely satisfied
Overall quality of the audio drama	◯	◯	◯	◯	◯
Content	◯	◯	◯	◯	◯
Plot/storyline	◯	◯	◯	◯	◯
Characters	◯	◯	◯	◯	◯
Sound	◯	◯	◯	◯	◯

	Extremely dissatisfied	Somewhat dissatisfied	Neither satisfied nor dissatisfied	Somewhat satisfied	Extremely satisfied
Voice acting	○	○	○	○	○
Easy to understand	○	○	○	○	○

Please share any additional comments or suggestions for improving the audio drama.

Pan feedback

Would you recommend this PAN.org.au to others?

- ○ Yes
- ○ No

What are your general impression of the PAN webstie?

	Strongly disagree	Somewhat disagree	Neither agree nor disagree	Somewhat agree	Strongly agree
I feel the content shown in the PAN website was relevant to me.	○	○	○	○	○
I learned information in the PAN website that will help me in preventing HIV.	○	○	○	○	○
I did not feel the PAN website gave me any information I did not already know.	○	○	○	○	○
I felt the content of the PAN website was culturally appropriate.	○	○	○	○	○
I think the content of the PAN website can help newly-arrived Asian born men better prevent HIV.	○	○	○	○	○
I think this PAN website is the best way to encourage newly-arrived Asian born men who have sex with men to learn about and start taking PrEP.	○	○	○	○	○

Your feedback on the PAN website will help us improve its quality and content.

	Extremely dissatisfied	Somewhat dissatisfied	Neither satisfied nor dissatisfied	Somewhat satisfied	Extremely satisfied
Overall quality of the PAN website	○	○	○	○	○
Content	○	○	○	○	○

	Extremely dissatisfied	Somewhat dissatisfied	Neither satisfied nor dissatisfied	Somewhat satisfied	Extremely satisfied
Appearance	○	○	○	○	○
Easy to navigate	○	○	○	○	○
Easy to understand	○	○	○	○	○

Please share any additional comments or suggestions for improving the PAN website.

Other comments

Any other comments?

End of survey

We thank you for your time spent taking this survey. You will receive an **AU$50 voucher** within the next 5 business days, assuming your answers are genuine.

You will receive **FURTHER AU$50 voucher** after completing two follow-up surveys at 4 and 8 weeks after completing this survey.

Powered by Qualtrics

Recaptcha

Before proceed, please
complete the captcha below.

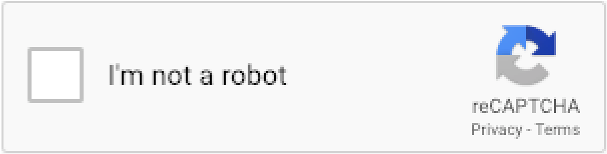

Eligibility

This is a four-week follow-up survey for the individual participating in the 'using online media to increase the awareness and uptake of PrEP among Asian-born men who have sex with men trial. In this survey, we would like to know the impact of our online media on your PrEP journey.

This survey will take approximately 5-10 minutes to complete.
You will receive AU$15 within 5 business days after completing the four week follow up survey and ,

assuming your answers deem genuine and detailed.

Enter the email address you registered to the trial

What was your participant ID?
(Please check the previous email you received from us for the participant ID)

What was the online media you received?

- ○ Hot Peach Tea Audio Drama
- ○ PAN.org.au Website

Survey

In the past month, have you considered taking PrEP?

- ○ Yes, I have
- ○ No, I have not

In the past month, have you taken any actions to start or use PrEP? (Tick all that apply)
(For example, I've shared PrEP informaiton with friend, seen a doctor about PrEP or purchased PrEP)

- ○ Yes, I have
- ○ No, I have not

What actions have you taken to start or use PrEP? (Tick all that apply)

- ☐ I have shared the audio drama/PAN.org.au with other people
- ☐ I have researched more about PrEP
- ☐ I have spoken to a doctor/nurse about PrEP
- ☐ I have gotten a HIV/STI test to start PrEP
- ☐ I have received PrEP prescription
- ☐ I have purchased PrEP
- ☐ I have started using PrEP
- ☐ I have started and stopped using PrEP

Can you share a copy of your doctor appointment confirmation, PrEP prescription, receipt, bottles or pills?
(JPEG, PDF, WORD are acceptable)

What factors have delayed your decision to start using PrEP in the past 4 weeks? (Please check all the apply)

- ☐ I'm worried about the side effects of PrEP.
- ☐ I'm worried about PrEP interacting with other medications or herbal medicines I'm taking.
- ☐ I don't think I need PrEP.
- ☐ I don't want other people to know that I'm on PrEP.
- ☐ I don't know how to access PrEP.
- ☐ I prefer to just use condoms to protect myself against HIV.
- ☐ I don't want to use my health insurance in case my parents find out I'm using PrEP.
- ☐ I find it too inconvenient to use PrEP.
- ☐ I can afford to use PrEP but I don't want to pay the high price.
- ☐ I cannot afford PrEP even though I want to use it.
- ☐ I don't know enough about PrEP
- ☐ Other (Please specify) ____________

What factors have influeneced your decision not to start using PrEP in the past 4 weeks? (Please check all the apply)

- ☐ I'm worried about the side effects of PrEP.
- ☐ I'm worried about PrEP interacting with other medications or herbal medicines I'm taking.
- ☐ I don't think I need PrEP.
- ☐ I don't want other people to know that I'm on PrEP.
- ☐ I don't know how to access PrEP.
- ☐ I prefer to just use condoms to protect myself against HIV.

- ☐ I don't want to use my health insurance in case my parents find out I'm using PrEP.
- ☐ I find it too inconvenient to use PrEP.
- ☐ I can afford to use PrEP but I don't want to pay the high price.
- ☐ I cannot afford PrEP even though I want to use it.
- ☐ I don't know enough about PrEP
- ☐ Other (Please specify) ________

Please explain your reasons for not starting PrEP below

____

How do you take PrEP?

- ○ Daily
- ○ On-demand or event-driven
- ○ Other (please specify) ________

How many PrEP pill have you taken since you started using it?

____

If you started PrEP, but stopped it, what factors have influenced your decision to stop?

Other comments

Any other comments?

End of Survey

Thank you for taking your time to complete the survey.
You will receive AU$15 within 5 business days after completing this survey, assuming your answers deem genuine.

You will receive additional AU$35 after completing an 8-week follow-up survey.

Powered by Qualtrics

Recaptcha

Before proceed, please
complete the captcha below.

☐ I'm not a robot

reCAPTCHA
Privacy - Terms

Eligibility

This is a eight-week follow-up survey for the individual participating in the 'using online media to increase the awareness and uptake of PrEP among Asian-born men who have sex with men trial. In this survey, we would like to know the impact of our online media on your PrEP journey.

This survey will take approximately 5-10 minutes to complete.
You will receive an AU$15 e-voucher within 5 business days after completing the eight week follow up survey and an $AU20 e-voucher if you completed all three surveys, assuming your answers deem

genuine and detailed.

Enter the email address you registered to the trial

What was your participant ID?
(Please check the previous email you received from us for the participant ID)

What was the online media you received?

- ○ Hot Peach Tea Audio Drama
- ○ PAN.org.au Website

Survey

In the past two months, have you considered taking PrEP?

- ○ Yes, I have
- ○ No, I have not

In the past two months, have you taken any actions to start or use PrEP? (Tick all that apply)
(For example, I've shared PrEP informaiton with friend, seen a doctor about PrEP or purchased PrEP)

- ○ Yes, I have
- ○ No, I have not

What actions have you taken to start or use PrEP? (Tick all that apply)

- ☐ I have shared the audio drama/PAN.org.au with other people
- ☐ I have researched more about PrEP
- ☐ I have spoken to a doctor/nurse about PrEP
- ☐ I have gotten a HIV/STI test to start PrEP
- ☐ I have received PrEP prescription
- ☐ I have purchased PrEP
- ☐ I have started using PrEP
- ☐ I have started and stopped using PrEP

Can you share a copy of your doctor appointment confirmation, PrEP prescription, receipt, bottles or pills?
(JPEG, PDF, WORD are acceptable)

What factors have delayed your decision to start using PrEP in the past 8 weeks? (Please check all the apply)

- [ ] I'm worried about the side effects of PrEP.
- [ ] I'm worried about PrEP interacting with other medications or herbal medicines I'm taking.
- [ ] I don't think I need PrEP.
- [ ] I don't want other people to know that I'm on PrEP.
- [ ] I don't know how to access PrEP.
- [ ] I prefer to just use condoms to protect myself against HIV.
- [ ] I don't want to use my health insurance in case my parents find out I'm using PrEP.
- [ ] I find it too inconvenient to use PrEP.
- [ ] I can afford to use PrEP but I don't want to pay the high price.
- [ ] I cannot afford PrEP even though I want to use it.
- [ ] I don't know enough about PrEP
- [ ] Other (Please specify) ____________

What factors have influeneced your decision not to start using PrEP in the past 8 weeks? (Please check all the apply)

- [ ] I'm worried about the side effects of PrEP.
- [ ] I'm worried about PrEP interacting with other medications or herbal medicines I'm taking.
- [ ] I don't think I need PrEP.
- [ ] I don't want other people to know that I'm on PrEP.

- [ ] I don't know how to access PrEP.
- [ ] I prefer to just use condoms to protect myself against HIV.
- [ ] I don't want to use my health insurance in case my parents find out I'm using PrEP.
- [ ] I find it too inconvenient to use PrEP.
- [ ] I can afford to use PrEP but I don't want to pay the high price.
- [ ] I cannot afford PrEP even though I want to use it.
- [ ] I don't know enough about PrEP
- [ ] Other (Please specify) ____________

Please explain your reasons for not starting PrEP below

____

How do you take PrEP?

- ○ Daily
- ○ On-demand or event-driven
- ○ Other (please specify) ____________

How many PrEP pill have you taken since you started using it?

____

If you started PrEP, but stopped it, what factors have influenced your decision to stop?

Other comments

Any other comments?

End of Survey

Thank you for taking your time to complete the survey.
You will receive AU$15 within 5 business days after completing this survey, assuming your answers deem genuine.

You will receive additional AU$35 after completing an 8-week follow-up survey.

Powered by Qualtrics
